# Synthetic Cell‐Based Tissues for Bottom‐Up Assembly of Artificial Lymphatic Organs

**DOI:** 10.1002/adhm.202503498

**Published:** 2025-10-23

**Authors:** Anna Burgstaller, Tamara Nink, Niklas Walter, Erick Angel Lopez Lopez, Hsin‐Fang Chang, Oskar Staufer

**Affiliations:** ^1^ INM – Leibniz Institute for New Materials Campus D2 2 66123 Saarbrücken Germany; ^2^ Helmholtz Institute for Pharmaceutical Research Saarland Helmholtz Center for Infection Research Campus E8 1 66123 Saarbrücken Germany; ^3^ Center for Biophysics Saarland University Campus Saarland 66123 Saarbrücken Germany; ^4^ Center for Integrative Physiology and Molecular Medicine (CIPMM) Saarland University 66421 Homburg Germany; ^5^ Max Planck Bristol Centre for Minimal Biology Cantock's Close Bristol BS8 1TS UK

**Keywords:** bottom‐up synthetic biology, immuno‐biophysics, regulatory T cells, synthetic cells, tissue engineering

## Abstract

Synthetic cells have emerged as a novel biomimetic approach for studying fundamental cellular functions and enabling new therapeutic interventions. However, the potential to program synthetic cells into self‐organized 3D collectives to replicate the structure and function of tissues has remained largely untapped. Here, self‐assembly properties are engineered into synthetic cells to form millimeter‐sized 3D lymphatic bottom‐up tissues (lymphBUTs) with mechanical adaptability, metabolic activity, and hierarchical microstructural organization. It is demonstrated that primary human immune cells spontaneously infiltrate and functionally integrate into these synthetic lymph nodes to form living tissue hybrids. Applying lymphBUTs, it is shown that structured 3D organization and mechanical support drives T cell activation and the application of lymphBUTs for ex vivo expansion of regulatory CD8^+^ T cells is demonstrated. The study highlights the functional integration of living and non‐living matter, advancing synthetic cell engineering toward 3D tissue structures.

## Graphical abstract

Synthetic cells have emerged as a novel biomimetic approach for fundamental research and therapeutic interventions. T cell activating synthetic cells are able to form 3D tissue‐like structures by self‐assembly into lymphatic bottom‐up tissues (lymphBUT) with tunable biochemical and biomechanical functionalities as well as metabolic activity are presented. Synthetic cells and human T cells can functionally integrate and form living tissue hybrids.

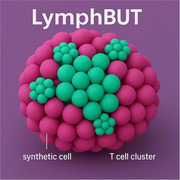

## 1 Introduction

Tissues are hierarchical cellular architectures formed from numerous different cell types with varying functionality. The organization and compartmentalization of tissues underlie cellular physiology and support the regulation of intercellular signaling. Translating this degree of mesoscale cellular organization to lab‐made biomaterials has remained a central ambition in designing biomimetics of functional tissue‐like structures.^[^ ^1^ ^]^ In this realm, bottom‐up synthetic biology opens the door to achieve the assembly of bionic cell‐like materials with defined and controlled structural and functional properties. In most implementations that applied synthetic cell engineering, individual dispersed synthetic cells, reminiscent of living singular cells, recreate a minimal metabolism, cell division, genome replication, or transcription and translation.^[^ ^2^, ^3^ ^]^ Commonly applied synthetic cell models include unilamellar vesicles, coacervates, colloidosomes, and microemulsions,^[^ ^4^, ^5^, ^6^ ^]^ each designed to mimic selected structural or functional features of living cells. However, based on these recent achievements in synthetic cell engineering, new opportunities arise to form whole synthetic cell‐based tissues with advanced therapeutic functionalities based on artificial cells.

While bottom‐up assembly of dispersed synthetic cells with biomedical value remains a formidable engineering challenge, recent pre‐clinical studies reported first successes of synthetic cells with therapeutic function, such as pro‐angiogenic effects, stimulation of neuronal stem cell differentiation, targeted cancer cell killing, and secretion of therapeutic proteins inside tumors.^[^ ^7^, ^8^, ^9^, ^10^ ^]^ Additionally, synthetic cell engineering enables major advancements in immunotherapy, specifically adoptive cell therapy (ACT), where they could be applied as advanced artificial antigen‐presenting cell (aAPC) models. Typically, ACT is based on the isolation of patient T cells, their expansion in an ex vivo environment and reinfusion into the patient after genetic modification.^[^ ^11^ ^]^ The efficiency of ACT relies on controlled ex vivo expansion of therapeutic T cell phenotypes that is achieved by ex vivo T cell cultivation with aAPCs in the form of solid dispersed polystyrene beads. These beads mimic the function of natural antigen presenting cells (APC) such as dendritic cells that provide crucial biochemical cues for T cell activation in vivo. aAPCs are typically decorated with antibodies directed against CD3 to induce T cell receptor (TCR) cross‐linking and CD28 activation for effective co‐stimulation. It has previously been shown that therapeutic T cell quality is influenced by more than biochemical signals provided by immune stimulating antibodies but also altered by biophysical factors such as synthetic cell size and shape, ligand mobility, rigidity, or viscoelasticity of aAPCs.^[^ ^12^, ^13^, ^14^ ^]^

In this regard, we previously developed an oil‐in‐water‐based dispersed synthetic cell model with a size of 3.75 µm (± 2.61 µm) formed from an elastomer core, surrounded by a lipid bilayer and stabilized by electrostatic interactions.^[^ ^15^ ^]^ This droplet supported lipid bilayers (dsLBs) technology can be adjusted in terms of elastic modulus ranging from 1.34 kPa (± 0.32 kPa) to 2990 kPa (± 8.94 kPa) and is therefore able to closely emulate the stiffness of natural T cell activating dendritic cells (≈3 kPa).^[^ ^16^ ^]^ The lateral mobile lipid membrane can be decorated with immune‐stimulating ligands at densities of 20 – 200 molecules µm^−2^ acting as aAPCs and therefore induce ex vivo T cell stimulation activation.^[^ ^15^ ^]^ Both, aAPC stiffness and ligand mobility on the aAPC membrane, are critical factors for the formation of signaling competent membrane–membrane interfaces, as required for the formation of supramolecular adhesion architectures in the T cells immune synapse.^[^ ^17^ ^]^

However, neither our dsLB‐based aAPC systems nor others based on solid beads or hydrogel particles do emulate the in vivo 3D tissue context of lymphatic organs, such as lymph nodes or spleen, in which T cells are naturally activated. Lymph nodes offer a physiological environment for T cell expansion, which has been shown to regulate the expansion behavior of T cells and contribute to their effector qualities.^[^ ^18^ ^]^

As 2D culture models can lead to morphological differences in cell shape and geometry as compared to in vivo‐resident cells, which can further result in altered gene expression and protein synthesis, the ACT field has been striving for alternative 3D culture models to integrate the tissue context for ex vivo T cell activation.^[^ ^19^ ^]^ For instance, recent studies have highlighted the relevance of the mechanical properties of 3D microenvironments for controlled ex vivo T cell expansion.^[^ ^20^, ^21^, ^22^ ^]^ Toward immunological tissue engineering Majedi et. al developed a 3D alginate‐based porous hydrogel with tunable rigidity and equipped with anti CD3/CD28 antibodies. Using this model, they showed varying activation dynamics of T cells induced by different scaffold rigidities and suggested that higher confined scaffolds lead to increased T cell activation.^[^ ^15^ ^]^ Furthermore, T cell cultivation and activation within a 3D scaffold have a positive impact on the T cell expansion compared to 2D models.^[^ ^23^ ^]^ Also, microfluidic‐generated alginate‐based aAPC microparticles decorated with immune stimulating antibodies together with mechanical forces provided by orbital shaking to T cells have been shown to increased TCR signaling, resulting in higher proliferation and the expansion of regulatory T cells emphasizing the importance of mechanical forces.^[^ ^24^ ^]^

In the context of synthetic tissue assembly from dispersed synthetic cells, structured 3D constructs built from interconnect synthetic cells have been formed from water‐in‐oil emulsions.^[^ ^25^ ^]^ Subsequently, bioprinting technologies were developed to form communicating 3D constructs based on aqueous droplet emulsions interconnected via lipid bilayers.^[^ ^26^ ^]^ However, the combination of tissue mechanics and APC biochemistry is central for immune cell expansion and therefore needs to be considered in order to design a relevant artificial immunological tissue model. As one initial approach, macroporous hydrogels have been equipped with bead‐based aAPC for in vivo expansion of engineered T cells resulting in enhanced antitumor efficacy.^[^ ^27^ ^]^

In summary, current ex vivo T cell activation systems are on the one hand limited by their 3D mechanical tissue‐like support and tissue‐like anatomical organization as they are only based on dispersed synthetic cell models. On the other hand, advanced models emphasizing tissue‐like 3D support are limited by the missing integration of physiologically relevant aAPC models.

Toward engineering fully synthetic cell‐based tissues with structured microanatomy of specialized biochemical and biomechanical functionalities, here we integrated dsLBs and vesicles to self‐assemble into artificial lymphatic organs. These advanced tissue mimetics combine biophysical properties and biochemical functionalities of natural tissue and integrate APCs in a 3D context focusing on tissue‐like structures rather than on the ECM. These lymphatic bottom‐up tissues (lymphBUTs) can provide primary human T cells with a synthetic cellular environment that supports their expansion upon activation. Functionality and structure observed in natural lymphatic tissues are integrated to study their impact on T cell differentiation and the efficiency of expansion. LymphBUTs therefore present the first synthetic cell‐based tissues, formed by self‐assembly from cell‐like bionic materials that sustain and develop the therapeutic functionality of human cells.

## 2 Results

### 2.1 LymphBUT Formation and Characterization

**Figure 1:**
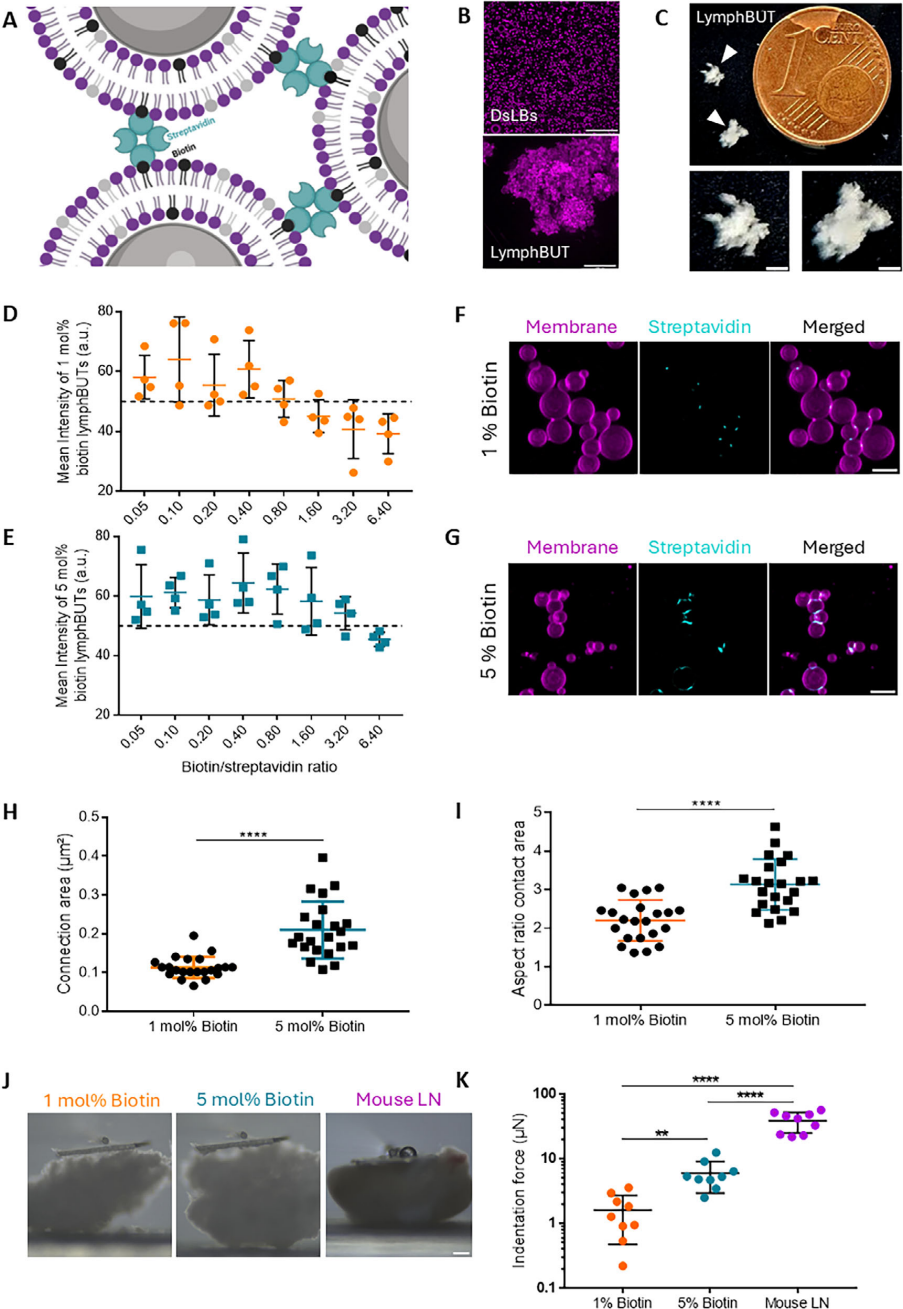
LymphBUT assembly and biomechanical characterization A) Schematic illustration of the self‐assembly process of dsLB‐based synthetic cells into lymphBUT mediated by biotin–streptavidin interactions. B) Representative confocal microscopy maximum z‐projections comparing disperse single dsLBs to a fully assembled lymphBUTs. Scale bars are 100 µm. C) Top view of two representative lymphBUTs (triangles) next to a 16.25 mm diameter 1 Eurocent for size comparison and lymphBUT magnifications. Scale bars are 1 mm. D,E) Stereomicroscopy imaging based mean intensity quantification of lymphBUTs connected via 1 mol% (D) or 5 mol% (E) biotin in the dsLB membrane and streptavidin at varying biotin: streptavidin molecular ratios (0.05/0.10/0.20/0.40/0.80/1.60/3.20/6.40:1). Dashed line indicates the threshold for fully formed lymphBUTs. The Mean intensity values are shown as mean ± SD from n = 4 separate lymphBUT batches. F,G) Representative confocal microscopy maximal z‐projections of lymphBUTs built from dsLBs with 1 mol% (F) and 5 mol% (G) biotin in the membrane connected by AlexaFluor405‐labeled streptavidin (1.40:1), concentrating at the connection sides between the dsLBs. Scale bars are 10 µm. HI) Quantification of connection area (H) and connection aspect ratio (I) from images in (F) and (G). Results are shown as mean ± SD from n = 22 manually segmented connection areas. *p* values were calculated using an unpaired two‐tailed student t test. J) Representative bright field images of 1 and 5 mol% biotin lymphBUTs and resected mouse lymph nodes in the micro‐indentation setup. Scale bar is 200 µm. K) Comparison of maximal indentation force required to compress 10% of total height of lymphBUTs made from dsLBs with 1 and 5 mol% biotin in the membrane and lymph nodes. Results are shown as mean ± SD from n = 9 lymphBUTs produced in two individual batches and two individual mouse lymph nodes n = 9. P values were calculated using an unpaired two‐tailed student t test. ^**^ *p* < 0.01, ^****^ *p* < 0.0001.

Toward efficiently forming synthetic cell‐based 3D tissues with adjustable mechanical properties, a dsLB‐based self‐assembly process was designed. The self‐assembly mechanism relies on interconnecting individual dsLBs by cross‐linking of biotin, integrated in the dsLB membrane via headgroup modified lipids, with tetrameric streptavidin (**Figure** **1**A). Through continuous orbital shaking (to promote mixing and synthetic cell contact formation) in a physiological buffer, macroscale tissue‐like structures, termed lymphBUTs, can be assembled with sizes between 1.0 and 4.0 mm (Figure 1B,C).

To investigate the impact of the streptavidin/biotin ratio on the lymphBUTs formation process and to potentially tune the mechanical properties of lymphBUTs, dsLBs with 1 and 5 mol% biotin in the membrane were formed. These were added to solutions of varying streptavidin concentration between 0.05:1 and 6.40:1 (biotin:streptavidin). Measuring the optical transmissibility to approximate lymphBUT density, we found that optimal ratios differ with varying biotin concentrations in the dsLB membrane (Figure 1D,E). DsLBs with 5 mol% biotin produced dense lymphBUTs at lower biotin/streptavidin ratios. This effect was observed over a broad range of streptavidin/biotin molecular ratio (0.05:1 to 3.20:1). At ratios above 0.05:1 we observed streptavidin saturation of the dsLB membranes and therefore no or only fragile lymphBUTs formation (Figure S1A, Supporting Information). Ratios below 0.80:1 for 1 mol% and 3.20:1 for 5 mol% biotin containing dsLBs did not show any lymphBUT formation. Of note, lymphBUTs could “self‐heal” after mechanical rupture by prolonged incubation in up to at least 5 formation‐destruction cycles without supplementation of further streptavidin (Figure S1B, Supporting Information). To investigate the mechanism behind the self‐healing effect, lymphBUTs were formed from two different dsLB populations (magenta (Rhodamine B) and green (Atto488) lipids). After mechanical disassembling and reformation, no fluorophore mixing in the dsLB membranes was observed. Therefore, we could exclude de‐ and reintegration of membrane lipids leading to the lymphBUT healing (Figure S1C, Supporting Information). Furthermore, adding AlexaFluor405‐labeled streptavidin to the lymphBUT fragments after mechanical rupturing revealed a newly formed streptavidin layer around the dsLBs. This confirmed residual accessible biotin in the dsLB membrane after lymphBUT formation (Figure S1D, Supporting Information). However, once in contact with biotin‐containing medium lymphBUT self‐healing is no longer possible based on the binding of the soluble biotin in the medium to the streptavidin in solution. Combining these results with results from Figure 1D,E, residual accessible biotin in the membrane and an excess of streptavidin in the supernatant is responsible for the lymphBUT self‐healing effect.

We next investigated the microstructural differences between 1 and 5 mol% lymphBUTs and imaged the connection areas between individual dsLBs by laser scanning confocal microscopy (LSCM) using AlexaFluor405‐labeled streptavidin. In both cases, we found streptavidin enrichment at the contact sites between individual dsLBs, however, with varying structure (Figure 1F,G). In 1 mol% biotin lymphBUTs, spot‐like connection clusters were observed, whereas 5 mol% biotin lymphBUTs showed significantly larger, more elongated planar connection areas (Figure 1H,I) and an increased streptavidin fluorescent intensity (Figure S1E, Supporting Information). This local enrichment also highlights the relevance of a laterally mobile membrane to sustain and support the formation of the supramolecular connection sites and the relevance of cell deformability to allow for planar interaction sites. Toward assessing whether the dsLB deformability is essential to the lymphBUT formation process and contact site maturation, we test synthetic tissue formation with lipid membrane‐coated silica beads of comparable size (3.94 µm) and identical membrane composition. We observed aggregation and initial contact formation between the beads in the center of the culture well, induced by the orbital shaking, but no 3D synthetic tissue formation (Figure S1G, Supporting Information). This indicates that a mobile membrane is not sufficient for stable contact formation, but a deformable synthetic cell is required to form robust intercellular adhesion interfaces as also observed in natural tissues.^[^ ^28^ ^]^ LymphBUT formation most likely necessitates the deformability of synthetic cells allowing the formation of robust planar adhesive interfaces.

From a macroscopic point of view, intercellular adhesion between artificial cells in tissues impacts the overall tissue mechanics. To evaluate if different dsLB interconnection architectures within lymphBUTs also translate to varying lymphBUT stiffnesses, we measured their compressibility by parallel plate compression analysis and compared the lymphBUT deformability to natural lymph nodes explanted from mice (Figure 1J). We compared the maximum indentation force needed to indent 10% of the total height of natural mouse lymph nodes or lymphBUTs formed from 1 and 5 mol% biotin dsLBs. The results demonstrate a significantly increased indentation force for 5 mol% biotin lymphBUTs compared to 1 mol% biotin lymphBUTs (Figure 1K), presumably based on the larger intercellular contact areas between dsLBs (see Figure 1F,G). Therefore, the lymphBUT tissue stiffness can be tuned by adjusting the inter‐dsLB connection sites via varying biotin concentrations, specifically between 1 and 5 mol%, while concentrations above 10 mol% did not result in a significant increase in stiffness. Of note, higher biotin concentrations in the dsLBs membrane between 10 and 30 mol% have been tested leading to irregular lymphBUT stiffnesses in the range of 5 mol% biotin lymphBUTs (Figure S1F, Supporting Information). Compared to natural similar‐sized mouse lymph nodes, 5 mol% lymphBUTs present a tenfold lower stiffness (Figure 1K). Since lymphBUTs intentionally only replicate the cellular components of tissues, not considering the ECM or the lymph node capsule, this difference in compressibility is reasonable and could in future be expanded with synthetic ECM components. As such, the lymphBUT system opens the door of synthetic cell‐based tissues with adjustable stiffness and biochemical composition properties.

### 2.2 Infiltration and Culture of Primary Human T Cells within LymphBUTs

**Figure 2:**
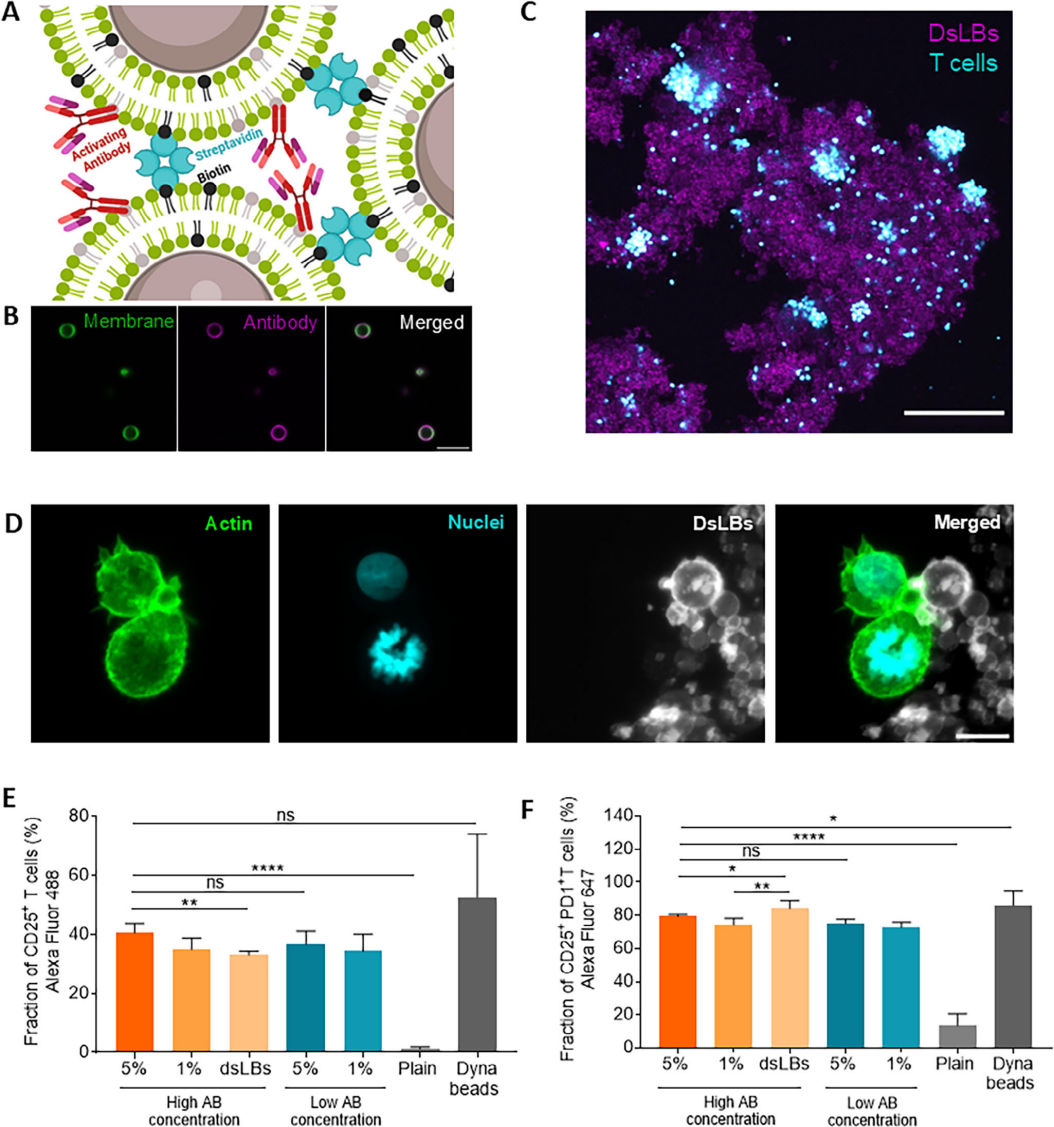
LymphBUT immune functionalization A) Schematic illustration of immune‐stimulating antibody decorated dsLBs interconnected by streptavidin and biotin. B) Representative confocal microscopy images of dsLBs functionalized with AlexFluor488‐labeled goat anti‐human IgG (magenta). Scale bar is 10 µm. C) Representative confocal microscopy maximal z‐projections showing human T cells (cyan) infiltrated into a 5 mol% biotin lymphBUT with a high anti‐CD3 and anti‐CD28 concentration (magenta). Scale bar 200 µm. D) Representative confocal microscopy maximal z‐projection of T cells (actin cytoskeleton = green, nucleus = cyan) in close contact with dsLBs (white) incorporated in lymphBUTs. Scale bar 5 µm. E,F) Quantification of the CD25^+^ (D) and CD25^+^ PD‐1^+^ (E) expressing T cell fraction expanded in different lymphBUT stiffnesses and antibody concentrations with dispersed dsLBs, Dynabeads or lymphBUTs that do not display antibodies (plain) measured by flow cytometry. Results are shown as mean ± SD of two donors in n > 2 technical replicates. *p* values were calculated using two‐tailed t test. ns = not significant *p* > 0.05, ^*^ *p* < 0.05, ^**^ *p* < 0.01, ^****^ *p* < 0.0001.

Toward activating and expanding T cells within lymphBUTs, we developed an approach for conjugating dsLBs with agonistic antibodies to induce T cell receptor activation (anti‐CD3) and T cell co‐stimulation (anti‐CD28)^[^ ^15^ ^],^ therefore creating aAPC from dsLBs. DsLBs were produced with a maleimide modified lipid membrane (final concentration of 5 mol%) to couple antibodies to their surface (**Figure** **2**A). LSCM imaging revealed a homogeneous distribution of AlexaFluor488‐labeled antibodies on the dsLB membrane and across the population (Figure 2B). We calibrated the surface density of the antibodies on the dsLB membrane via beads of known molecules of equivalent soluble fluorochrome (MESF) (Figure S2A, Supporting Information). In further experiments, a 1:3 ratio of anti‐CD3 and anti‐CD28 antibodies was used at a final density of 1020 molecules µm^−2^ (high) or 786 molecules µm^−2^ (low). Importantly, when incubating CD8^+^ T cells with lymphBUTs, we observed their spontaneous infiltration into the lymphBUTs, in the sense that T cells were not injected or forced in any other way to enter the lymphBUTs. The T cell migration was observed by live cell tracking of the Hoechst33342 stained CD8^+^ T cell nuclei for 15 h using LSCM. We found that T cell migrated mainly within cavities of the lymphBUTs at different speeds continuously interacting with individual dsLBs (Movie S1, Supporting Information). Importantly, we also observed that T cells undergo transient phases of high confinement when migrating through tiny channels within the lymphBUT architecture indicated by the transiently deformed cell nuclei. The cell nuclei also indicated the state of the cell cycle by their sizes (Figure S2B, Supporting Information). After 4 days of incubation, T cells formed confined activation clusters reminiscent of clonal expansion clusters in lymph nodes^[^ ^29^ ^]^ (Figure 2C). LSCM imaging showed T cells being in close contact and forming immunological synapses with antibody‐decorated dsLBs that act as aAPC within the lymphBUT (Figure 2D). We quantified the activated T cells cultivated within lymphBUTs of different densities and antibody concentrations by staining for CD25 expression. Irrespectively from the lymphBUT density and the antibody concentrations, the CD25 expression was between 32% and 40% of the total T cell population cultivated with lymphBUTs. As we have previously investigated, the lateral mobility of antibodies on the lipid membrane surface of dsLBs masks antibody concentration effects. This is supported by confocal microscopy imaging that showed antibody clustering at the interface between T cells and dsLBs (14). Therefore, while overall antibody density is reduced in the “low” condition, based on lateral aggregation on the T cell contact side, density effects play a reduced role in the dsLB system. However, the CD25 expression of T cells activated within lymphBUTs was lower than for T cells activated with the industry standard Dynabeads (Figure 2E). Interestingly, when quantifying the expression of the immune‐checkpoint receptor PD‐1 among the CD25^+^ T cells, we found that T cells expanded within lymphBUTs expressed a significantly reduced fraction of PD‐1^+^ cells as compared to dispersed dsLBs and Dynabeads (Figure 2F). This indicates that the combination of biomechanical support provided by lymphBUTs could expand T cells with an improved phenotype that is less prone to immune suppression.

### 2.3 Formation of Heterotypic LymphBUTs with Spatially Organized Functional Zones

**Figure 3:**
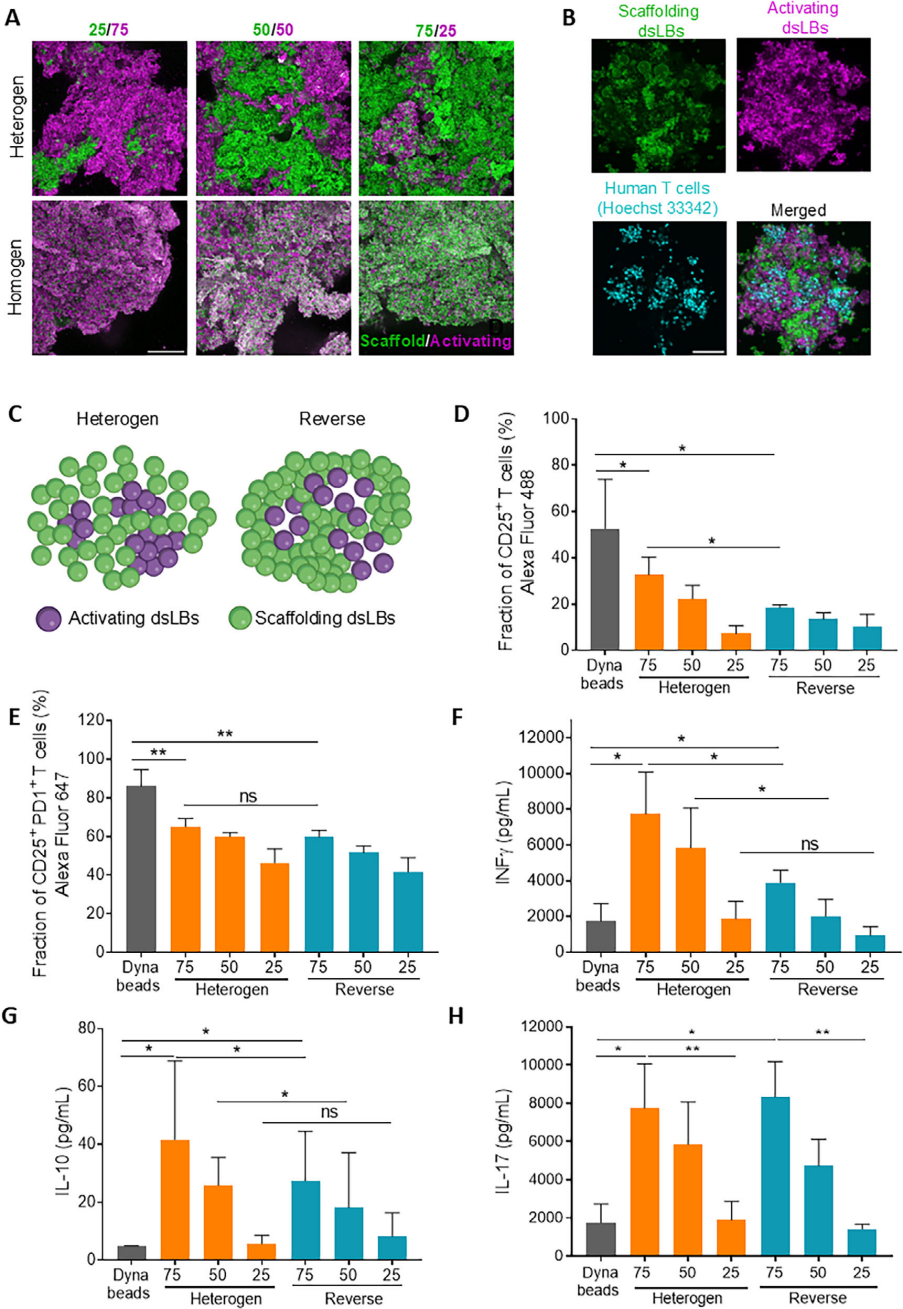
Heterogenous tissue formation and functionality A) Representative confocal microscopy maximal z‐projection of hetero‐(top) and homogeneous (bottom) lymphBUTs formed from different ratios of activating dsLBs (magenta) and scaffolding dsLBs (green). Scale bar is 100 µm. B) Representative confocal microscopy maximal z‐projection of a heterogeneous lymphBUT with immune‐activation zones (magenta) and scaffold areas (green), incubated with human CD8^+^ T cells for 4 days and stained with Hoechst 33342 (cyan). The T cells expanded within the lymphBUT after 4 days of co‐cultivation. Scale bar is 100 µm. C) Schematic illustrations showing different lymphBUT architectures (heterogeneous and reverse) assembled from 5 mol% and 1 mol% biotin dsLBs displaying no (green) or a high anti‐CD3 and anti‐CD28 concentration (magenta). D) and E) Flow cytometry quantification of CD25^+^ CD8^+^ T cell population (D) and CD25^+^ PD‐1^+^ T cell subpopulation (E). F) Quantification of INFγ cytokine release via an automated ELISA. G) and H) Phenotyping of human CD8^+^ T cells by analyzing the IL10 (G) and the IL‐17 (H) cytokine profile. Comparison of heterogeneous and reverse lymphBUTs with varying ratios of activating and non‐activating dsLBs (75%, 50%, 25%) displaying a high anti‐CD3 and anti‐CD28 concentration with Dynabeads. Results are shown as mean ± SD of two donors n > 2. *p* values were calculated using two‐tailed t test. ns = not significant *p* > 0.05, ^*^ *p* < 0.05, ^**^ *p* < 0.01, ^***^ *p* < 0.001, ^****^ *p* < 0.0001.

Lymph nodes, like many other tissues, are organized in a hierarchical structure with spatially segregated tissue functions (e.g., T cell zones, germinal centers). This architecture is crucial to separate effector and regulatory cells and provides a mechanical environment for expansion. For instance, fibroblasts in the lymph node stroma provide mechanical support for lymph node swelling and controlled T cell expansion.^[^ ^30^ ^]^ Toward mimicking this multifunctional heterotypic cellular architecture with varying biochemical and biomechanical properties in a simplified manner, we implemented a sequential formation process to build spatial heterogenous lymphBUTs taking advantage of the lymphBUT self‐healing properties (Figure S1C, Supporting Information). In a first step, lymphBUTs are formed from antibody‐decorated, T cell‐activating dsLBs and homogenized by resuspension into smaller lymphBUTs of ≈50 – 100 µm size. In analogy to the dense dendritic cell network in T cell zones of lymph nodes, dsLBs with 5 mol% biotin were used for this initial assembly step. Optical analysis of the density of lymphBUTs formed with different biotin concentrations (see Figure 1D,E) as well as confocal microscopy observations showed that lymphBUTs with 5 mol% biotin form a substantially denser dsLB network, presumably translating to a higher degree of T cell confinement in these zones. By subsequently adding scaffolding (non‐functionalized) dsLBs, a scaffold is formed connecting the activating small lymphBUTs. This loser network made of 1 mol% dsLBs is able to expand and swell as a response to intra‐lymphBUT T cell expansion. LSCM imaging confirmed the formation of heterotypic lymphBUTs containing distinct stimulatory zones (magenta), surrounded by a non‐stimulatory scaffold (green). We successfully formed heterotypic lymphBUTs with varying ratios of reaction zones to scaffold (75/25, 50/50, 25/75) showing segregated architectures as compared to homogeneous lymphBUTs formed from pre‐mixed activating (magenta) and scaffolding (green) dsLBs (**Figure** **3**A). This demonstrates that the hierarchical architecture of natural tissues can be mirrored to a simplified degree with lymphBUTs.

To assess directed migration of T cells into the activating zones of heterotypic lymphBUTs, we performed LSCM imaging of T cell infiltrated into the lymphBUTs after 4 days of co‐cultivation and found T cell expansion clusters prevalently located in the activating areas rather than the scaffold (Figure 3B). To quantify the localization of T cells in the heterotypic lymphBUTs, co‐localization analysis of the cells with the distinct lymphBUT zones was performed, showing a significantly increased T cell cluster appearance in the activation zones compared to the scaffolding zones (Figure S2D, Supporting Information). This was also the case in “reverse lymphBUTs” where the activation zones were formed from less dense 1 mol% biotin dsLBs and the scaffold by the denser 5 mol% dsLBs. These results indicate that the localization of T cells is mainly driven by the biochemical signals presented in the activating zones rather than the zone density.

### 2.4 Characterization of T Cell Phenotypes Cultured in LymphBUTs

Toward quantifying the relevance of lymphBUT heterogeneity and density for T cell expansion, we next measured expression markers and cytokine profiles of T cells incubated in the various lymphBUT configurations, including the reverse lymphBUTs, and compared these to Dynabeads (Figure 3C). Quantification of CD25^+^ T cells showed the highest T cell activation by Dynabeads, followed by 75 (75% activating and 25% scaffolding dsLBs) heterogenous lymphBUTs (Figure 3D), while reverse lymphBUTs have a significantly lower ability to activate T cells compared to the heterotypic and homogenous conditions (Figure S3A, Supporting Information). This highlights the relevance of a looser scaffold serving as expansion matrix for T cell activation. For all conditions, a clear trend in CD25 expression depending on the concentration of activating dsLBs was observed. A similar trend was detected when quantifying CD25^+^ PD‐1^+^ T cells. PD‐1 is an immune‐suppression receptor that is usually upregulated by state‐of‐the‐art ex vivo T cell expansion technologies. This upregulation diminishes the ability of cytokine secretion and is disadvantageous for successful T cell therapy.^[^ ^31^ ^]^ We confirmed a high fraction of CD25^+^ PD‐1^+^ T cells activated with Dynabeads. Importantly, the PD‐1 expression was significantly reduced for lymphBUT‐expanded T cells (Figure 3E; Figure S3B, Supporting Information). Interestingly, we also measured a significantly increased release of INFγ by T cells activated in lymphBUTs as compared to Dynabeads activated ones (Figure 3F; Figure S3C, Supporting Information). The effector cytokine INFγ has been controversially discussed for its tumor promoting and suppressing capabilities^[^ ^32^ ^]^ but is critical for the efficacy of cytotoxic T cell in ACT.^[^ ^33^ ^]^ This shows that while the overall activation efficacy (CD25) is lower in lymphBUTs, the expression of immuno‐suppressive receptors (PD‐1) and the secretion of effector cytokines (INFγ) is differentially regulated in the 3D expansion matrix.

This observation was further supported by the quantification of the cytokines IL‐10 and IL‐17 as well as the expression of CD103 expression, a tissue resident T cell marker (Figure 3G,F; Figure S3D, Supporting Information). Contrary, the release of the pro‐inflammatory cytokine IL‐17 and the regulatory cytokine IL‐10 is upregulated in T cells stimulated with lymphBUTs compared to Dynabead‐expanded T cells (Figure 3H; Figure S3E,F, Supporting Information). Again, the lower the concentration of activating dsLBs incorporated into lymphBUTs the lower the cytokine concentrations detected. Of note, activating T cells with disperse dsLBs changed the secretion of IL‐10, IL‐17, and INFγ to be closer to dispersed Dynabeads as to dsLB‐based lymphBUTs, indicating that the observed differences can be mostly attributed to the 3D setting rather than to the difference in the aAPC technology (Figure S3A,C,E,F, Supporting Information). These results suggest that considerable T cell activation can be achieved with the lymphBUT technology, offering a platform for expansion of a distinct CD8^+^ regulatory‐like T cell phenotype as compared to monodisperse systems including the commercial gold standard Dynabeads.

**Figure 4:**
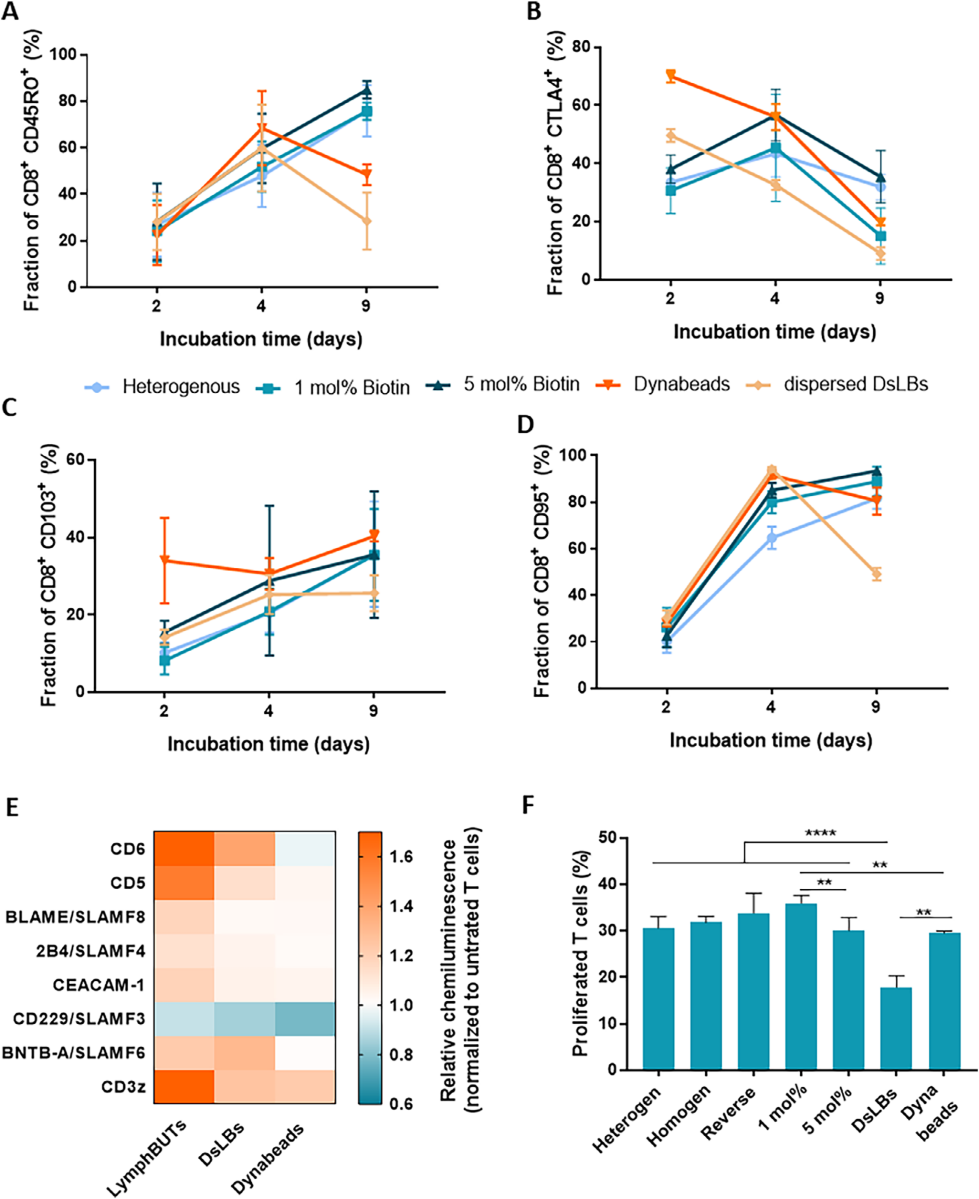
CD8^+^ T cell phenotyping and proliferation A–D) Phenotyping of human CD8^+^ T cells by quantifying CD45RO^+^ (A), CTLA4^+^ (B), CD103^+^ (C) and CD95^+^ (D) T cell subpopulations via flow cytometry comparing the receptor expression of T cells cultivated with heterogenous, loose 1 mol% biotin and dense 5 mol% biotin lymphBUTs as well as with Dynabeads and dispersed dsLBs for 2, 4, and 9 days. Results are shown as mean ± SD of two donors n = 2. E) Analyzing the phosphorylation status of eight different proteins compared between lymphBUTs, dsLBs, and Dynabeads using Proteom Profiler. Results are shown as mean ± SD of four donors. F) Comparison of the proliferated T cell fraction between different lymphBUT conditions (heterogeny, homogeneous, reverse, 1 mol% biotin, 5 moil% biotin) as well as dsLBs and Dynabeads quantifying the CellTrace CFSE stained T cells via flow cytometry. Results are shown as mean ± SD of two donors n = 2. *p* values were calculated using one‐way ANOVA. ns = not significant *p* > 0.05, ^*^ *p* < 0.05, ^**^ *p* < 0.01, ^***^ *p* < 0.001, ^****^ *p* < 0.0001.

To further investigate the impact of a 3D tissue mimicking environments on the T cell phenotype, we quantified expression of relevant T cell surface receptors (CD45RO, CD103, CTLA4, and CD95) after 2, 4, and 9 days of activation. CD45RO expression is not only associated with a memory phenotype but also with a positive prognostic effect for solid tumors in ACT.^[^ ^34^ ^]^ We observed that the fraction of CD8^+^ CD45RO^+^ T cells evolved in a time‐dependent manner. While initial dynamics of CD45RO^+^ expression were similar among all activation approaches, only lymphBUT activated T cells and none of the dispersed systems (Dynabeads or dsLBs) continued to produce CD45RO^+^ cells (**Figure** **4**A). T cells cultivated with dispersed aAPC systems showed a high CTLA4 expression after 2 days of 50% for dsLBs and 70% for Dynabeads that decreased continuously toward 10–20% CTLA^+^ T cells. While 3D lymphBUT cultivated T cells had a lower CTLA4^+^ T cell population after 2 days of culture of about 30–40%, this increased after 4 days before it decreased again (Figure 4B). Similar differences in expression dynamics could also be observed for CD103 and CD95 (Figure 4C,D), all together pointing to a underlaying different activation dynamic of T cells between dispersed aAPCs and 3D lymphBUT systems and resulting in a different phenotype after activation for longer time periods.

Of note, in addition to isolated CD8^+^ T cells we also resolved the effects when immune cell collectives were cultured within lymphBUTs. For this, Peripheral Blood Mononuclear Cells (PBMC) were isolated, cultivated for 4 days with diverse lymphBUT conditions as well as dispersed dsLBs and Dynabeads and analyzed with a flow cytometry phenotype panel (Figure S4A–F, Supporting Information). By integrating a greater diversity of cell types, we observed differences compared to isolated CD8^+^ T cell cultures, most likely through additional intercellular communication via cytokines and other small molecules. Strong effects could be detected when measuring the surface receptors CCR7, CD103, and especially CD62L and CD122. While CD4^+^ T cells express higher levels of CCR7 and CD45RO they showed reduced expression of CD103 and CD122 compared to CD8^+^ T cells in the PBMC mix. Interestingly, the 3D properties of lymphBUTs have a major effect on CTLA4 expression of T cell within the PBMC mix and increased the CTLA4^+^ T cell fraction in lymphBUT cultivated T cells. Overall, when applying PBMC cultures, smaller differences in marker expression between individual lymphBUT configurations could be observed.

We next aimed to resolve differences in the signaling mechanisms between the individual activation technologies by proteome profiling. This method allows a semi quantitative measurement of phosphorylated signaling proteins involved in the T cell activation process. The differences between 3D lymphBUTs and dispersed dsLBs and Dynabeads could be confirmed (Figure 4E). While dsLBs and Dynabeads show a similar phosphorylation degree of CD3z, indicating T cell activation, lymphBUT cultivated T cells demonstrate an increased phosphorylation status. At the same time, phosphorylation of the co‐regulatory receptors CD6 and CD5 was lower in T cells stimulated with Dynabeads compared to T cells activated with dsLBs and lymphBUTs. CD5 and CD6 are involved in immunological synapse formation and modulate TCR signaling, also supporting the establishment of adhesive interfaces.^[^ ^35^, ^36^, ^37^ ^]^ The reduced phosphorylation observed with Dynabeads likely reflects their rigidity and the absence of laterally mobile ligands, in contrast to the more membrane‐like properties of dsLBs and lymphBUTs. Similar patterns could be detected for the Signaling Lymphocyte Activation Molecule Family (SLAMF). On the other hand, CD229/SLAMF3 showed a reversed effect. These results do not just suggest a clear change in the phosphorylation pattern after activation between dispersed and 3D activation techniques, but also aAPC elasticity and membrane properties impacting the T cell phenotype correlating with results shown in Figure S3A,C,E,F (Supporting Information).

Next, the fraction of proliferated T cells after 4 days of co‐cultivation with different lymphBUT architectures (heterogeny, homogen, reverse, 1 mol% biotin, and 5 mol% biotin), dispersed dsLBs and Dynabeads were quantified and compared. The T cell proliferation is significantly increased between dispersed dsLBs and all lymphBUT conditions and Dynabeads as well as between Dynabeads and loose 1 mol% lymphBUTs. Comparing the individual lymphBUT architectures, only minor differences could be observed with the exception of loose 1 mol% and dense 5 mol% lymphBUTs, again highlighting the relevance of synthetic tissue confinement and density (Figure 4F).

### 2.5 Integrating Metabolic Active Synthetic Cells into LymphBUTs

**Figure 5:**
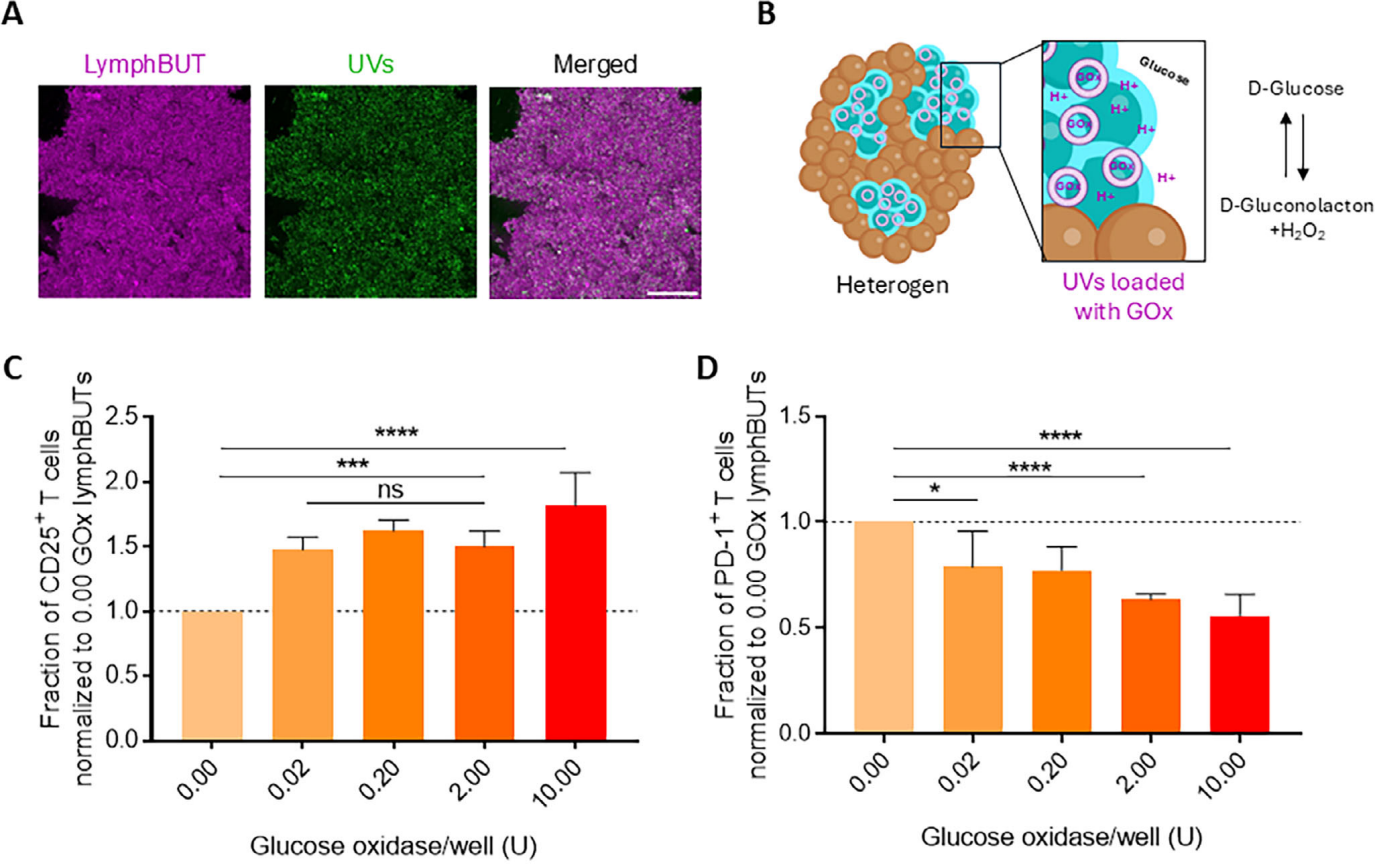
Integration of metabolic active components within lymphBUTs. A) Representative confocal microscopy image of UVs incorporated within a lymphBUT. Scale bar is 100 µm. B) Schematic illustration of glucose oxidase loaded UVs incorporated in the T cell activation zones of 75% heterogeneous lymphBUTs. C,D) Flow cytometry quantification of CD25^+^ CD8^+^ (C) and PD‐1+ CD8+ (D) T cell populations dependent on the UV concentration and therefore Glucose oxidase concentration in lymphBUTs (75% heterotypic). Results are shown as mean ± SD of two donors n = 2. P values were calculated using one‐way ANOVA. ns = not significant *p* > 0.05, ^*^ *p* < 0.05, ^**^ *p* < 0.01, ^****^ *p* < 0.0001.

Natural tissues do not only regulate physiological functions via structural and mechanical properties but also provide an appropriate metabolic milieu. For instance, dendritic cells reduce the local pH in lymph nodes to sustain T cell differentiation and secrete H_2_O_2_ to further boost T cell expansion.^[^ ^38^ ^]^ Toward integrating these metabolic aspects into lymphBUTs, we introduced metabolically active synthetic cells in the form of enzyme‐loaded unilamellar vesicles (UVs). LSCM confirmed the integration of a varying number of UVs, containing biotin‐modified lipids in their membrane, into lymphBUTs (**Figure** **5**A; Figure S2E, Supporting Information), which homogeneously distributed within the lymphBUT. To mimic the pH reduction and H_2_O_2_ production by dendritic cells, we loaded the UVs with glucose oxidase (GOx) enzymes and produced the UV with a membrane composition that allows for passive diffusion of glucose but retention of the enzyme.^[^ ^39^ ^]^ GOx reduces the culture pH via glucose oxidation and produces H_2_O_2_ as a side product (Figure 5B).

By tuning the number of GOx‐loaded UVs integrated into the 75/25 heterogeneous lymphBUTs, we produced synthetic tissues with 0.00, 0.02, 0.20, 2.00, and 10.00 U GOx and cultivated these with primary human CD8^+^ T cells. The effect of the GOx‐modulated microenvironment within the synthetic tissues on T cell activation, as quantified by CD25 expression, and the immunosuppressive susceptibility, as assessed by PD‐1 quantification, was measured. We could resolve a significant increase in T cell activation shown by the CD25^+^ T cell fraction when integrating metabolic UVs into the lymphBUTs (Figure 5C). Interestingly, while the CD25 signal increased, the PD‐1 signal gradually decreased in a GOx‐dependent manner. This indicates a positive effect of GOx integrated into 3D structures on the T cells' susceptibility for immune suppression after 4 days of activation. Taking together, these results demonstrate that lymphBUTs are not only able to replicate tissue structure and mechanical dynamics but also integrate key biochemical triggers relevant to tune T cell expansion.

### 2.6 Summary and Outlook

In this study, we present a bottom‐up synthetic biology inspired system for synthetic tissue assembly, meeting a biomedical need in immunotherapy. LymphBUTs are based on dispersed synthetic cells, engineered to self‐assemble, adding a third and macroscale dimension to synthetic cell engineering.^[^ ^4^ ^]^ LymphBUTs reach sizes of 1–4 mm and therefore mimic structural features of many natural tissues, including lymph nodes, follicles, taste buds, or Langerhans islets. Their overall and local density, architecture as well as their immunological and metabolic properties can be adjusted to mimic the structure and function of natural lymphatic tissues in a reduction of complexity approach.

We showcase their application for expansion of distinct T cell phenotypes in lymph node‐mimicking environments. Previous technologies for this are either based on monodispersed bead systems, focusing on receptor stimulation, or artificial 3D matrices integrating a chemical stimulus into bio‐printed isotropic bulk materials.^[^ ^10^ ^]^ In contrast, lymphBUTs can be formed with spatially segregated T cell activation zones, comprised of dense immune activating artificial antigen presenting cells, surrounded by an expandable scaffolding tissue and functionalized with metabolically active synthetic cells. T cells infiltrate the lymphBUTs and preferentially activate and form clusters within the T cell activation zone independent of the activation zone density. With this, the synthetic tissues formed in lymphBUTs recreate three distinct features of tissues: Mechanical adaptability, metabolic activity, and microstructural hierarchical organization.^[^ ^40^ ^]^

We benchmarked the lymphBUT technology to commercial ex vivo T cell activation beads (Dynabeads) as they are commonly applied in many clinical applications and academic labs due to their fast activation and commercial availability. Flow cytometry and secretome results revealed the expression of a distinct regulatory‐like CD8*^+^* T cell phenotype characterized by a high INFγ release as well as IL‐10 secretion. This phenotype has already been reported and initial applicability for immunotherapeutic approaches has been suggested.^[^ ^16^ ^]^ Even though the T cell activation measured by the CD25 signal is slightly lower on T cells cultivated with lymphBUTs as compared to Dynabeads, they showed a significantly reduced PD‐1 expression, indicating a phenotype that is less prone to immune suppression. Cheung et al. developed mesoporous silica micro‐rods with a mobile lipid membrane and introducible metabolic activity. Their results also indicated a T cell population after activation that is less prone to immune suppression shown by a reduced PD‐1 signal indicating that PD‐1 expression is mainly driven by the mobility of the immune stimulating receptors on the aAPC surface.^[^ ^15^, ^41^ ^]^ Interestingly, we observed differences in the activation dynamics comparing individual lymphBUT architectures. In natural lymphatic tissue, the reaction areas, e.g., T cell zones or germinal center, host the majority of immune cells and feature dense networks of dendritic cells. In analogy to natural lymph nodes, lymphBUTs with denser activation zones and looser scaffolds were designed. By comparing T cell expansion in these lymphBUTs with reverse lymphBUTs we could show that this structure is crucial for efficient T cell expansion and demonstrate the bottom‐up formation of a synthetic cell‐based tissue with functional microanatomy.

Therefore, we successfully applied a synthetic biology‐driven reduction of complexity approach to introduce structural cellular hierarchy in synthetic tissue. The lymphBUT system is one of the first approaches for synthetic and natural cell hybrids and therefore opens a new door for future applications of synthetic cells in biomedicine. In the future perspective, we aim to further leverage the major advantage of synthetic biology of controllability to not only tune lymphBUT size and stiffness but also single dsLB arrangement and lymphBUT morphology. Furthermore, the lymphBUT technology is not limited by immune cell co‐cultivation for immunotherapeutic applications but can be applied as a novel cell culture system for any other system that currently requires feeder cells or other support structures. So, it also provides a platform to mimic other tissue structures such as neurological tissue or hormone glands and study the tissue signaling process in health and disease under defined molecular control in 3D. Since lymphBUTs allow for a high degree of flexibility in various aspects, while at the same time they can be produced in a controlled manner in high throughput format, they carry immense potential for further applications such as drug screening or a matrix for toxicity tests.

## 3 Experimental Section

### DsLB Production

DsLBs were produced using electrostatic interactions in a layer‐by‐layer formation approach based on an oil in water emulsion. For that 100 mg of PDMS (Sylgard 184, Dow Corning USA) were pre‐emulsified by resuspension in 910 µL of PBS adjusted to pH 7 with HCl and containing 4 mm of Sodium dodecyl sulfate (SDS, Sigma–Aldrich, Germany)). The oil in water droplets was emulsified for 2 min using a sonification bath. In the next step, 22 mm of MgCl_2_ (Sigma–Aldrich, Germany) and 100 µL of 6 mm small unilamellar vesicles (SUVs) were added and the mixture was incubated for 2 min protected from light. The excess SUVs were removed by centrifugation at 10 000 xg for 30 s, discharging of the supernatant and resuspension in 1 mL PBS adjusted to pH 7 with HCl. This washing step was repeated for a second time.

The SUVs were produced using extrusion as described previously.^[^ ^15^ ^]^ They consist of 20 mol% EggPG, 5 mol% 18:1 MPB PE (maleimide), 1 or 5 mol% 18:1 Biotinyl PE (biotin), 1 mol% LissRhodamine B‐PE, Atto488‐PE or 18:0 Cy5 PE, and EggPC acting as a filling lipid (all Avanti Polar Lipids, USA).

### DsLB Immune Functionalization

To integrate immune activating properties within the dsLB membrane a functionalized lipid equipped with a maleimide headgroup (Avanti Polar Lipids, USA) was integrated with 5 mol%. The maleimide headgroup can form a thiol connection with cysteine residues located on antibodies and proteins. For this antibody decoration, the total accessible amount of maleimide of the dsLB suspension needs to be determined. In order to do so, a SUV 1:1 dilution series ranging from 60 to 0.47 µm was prepared in a 96‐well plate and used for calibration. The dsLB suspension was diluted 1:10 with PBS into the well plate.

The fluorescent intensity was measured with a TECAN Spark plate reader (Tecan Group, Switzerland) controlled by TECAN SparkControl software with in‐built gain optimization. The excitation/emission settings were adjusted to Rhodamine B (537/582 nm), Atto488‐PE (495/545 nm), or CY5‐PE (630/675 nm) labeled dsLBs.

Based on the total lipid membrane concentration, the dsLBs were incubated with immune‐stimulating antibodies against human CD3 (UCHT1, Invitrogen) and CD28 (CD28.2, Invitrogen) at a ratio (maleimide/antibody) of 0.5 for the high antibody condition and 0.125 for the low antibody condition and an adjusted αCD3/αCD28 ratio of 1:3. Before adding the antibody mix to the dsLBs, the antibodies were washed using the MircoSpin G‐25 Columns (Cytiva, US) according to manufacturer instructions. The antibody yield of 91% (±1%) was considered for the calculation (data not shown). The dsLBs were incubated with the antibodies in PBS adjusted to pH 7 at RT for 1 h resuspending every 15 min. The incubation was followed by a washing step. In order to know the dsLB concentration, the total lipid concentration was determined again by measuring the fluorescent intensity with the plate reader as already described.

### MESF Bead Assay

To quantify the antibody concentration on the dsLB membrane, a quantitative image‐based Molecules of Equivalent Soluble Fluorochrome (MESF) assay was performed. For that dsLBs with 5 mol% maleimide in the membrane were produced and the lipid concentration was determined as described in dsLB immune functionalization. Based on the lipid concentration, the accessible maleimide concentration was calculated. The dsLBs were functionalized with different ratios of maleimide/Alexa Fluor 488 anit‐CD3 (HIT3a, BioLegend, UK) (1:1, 1:2, 1:4, 1:8, 1:16, 1:32). The Alexa Fluor 488 anit‐CD3 was cleaned using the MircoSpin G‐25 Columns (Cytiva, US) according to manufacturer instructions and the antibody yield of 91% (±1%) was considered within the calculations. The mixture was incubated in PBS pH 7 for 1 h at RT protected from light and resuspended gently every 15 min. After the incubation the dsLBs were centrifuged at 10 000 xg for 30 s and resuspended in 200 µL PBS + 1% BSA. The antibody decorated dsLBs were transferred to an 8‐well Nunc LabTeK glass bottom chamber with a mixture of Quantum Alexa 488 MESF beads (Bangs Laboratories Inc., USA) according to manufacturer instructions. The samples as well as the 488 MESF beads were imaged using a laser scanning microscope LSM 880 (Carl Zeiss AB) equipped with 20x objective (Plan‐Apochromat 20x/0.8 M27, Carl Zeiss AG, Germany) and a 488 nm laser line. The analysis was performed with the ImageJ software (NIH, USA) by background subtraction, global‐threshold segmentation, fill hole function, watershed particle separation, and automated particle detection, measuring the fluorescent intensity of disperse dsLBs. The calibration was based on the four bead populations with known MESF/bead correlated to their fluorescent intensities. For calculating the antibody density per µm^2^ based on the MESF/area on dsLBs the fluorophores/antibody ratio was determined with the help of the NanoDrop (Thermo Fisher, Germany).

### LymphBUT Formation Based on dsLBs

The incorporation of biotinylated lipids within the dsLB membrane combined with external provided streptavidin allows the self‐assembly of lymphBUTs. For this purpose, dsLBs were produced as already described with additional 1 or 5 mol% Biotin (Avanti Polar Lipids, USA) in the lipid membrane. In order to form lymphBUTs of about a similar size and number of dsLBs, the total lipid concentration of the dsLB membrane was determined as described above by measuring the fluorescent intensity using the TECAN Spark plate reader (Tecan Group, Switzerland). A total number of ≈2.7 million dsLBs were transferred to a 48‐well plate. These wells were filled with PBS to a final volume of 400 – 450 µL containing streptavidin (Invitrogen, USA) in a molecular biotin/streptavidin ratio of 0.05, 0.10, 0.20, 0.40, 0.80, 1.60, 3.20, or 6.40:1. By orbital shaking at 450 rpm for 1–2 h the biotin was exposed to the diffusing streptavidin leading to the lymphBUT self‐assembly.

### LymphBUT Architectures

The lymphBUT formation strategy based on a strong connection between biotin and streptavidin allows the engineering of diverse lymphBUT architectures. LymphBUTs can be self‐assembled from dsLBs displaying no T cell activating antibodies (plain). Contrary, they can be formed of dsLBs decorated with immune‐stimulating antibodies against human CD3 (UCHT1, Invitrogen) and CD28 (CD28.2, Invitrogen) in a 1 to 3 ratio and high (1020 molecules µm^−2^) or low antibody conditions (786 molecules µm^−2^).

In order to achieve a hierarchical structure within a heterotypic lymphBUT a sequential formation procedure was developed. In the first step, a lymphBUT was formed from T cell activating, high antibody decorated (activating) 5 mol% biotin dsLBs using a biotin/streptavidin ratio of 0.40:1 in 48‐well plates with a final PBS volume of 400 µL. After 1 h of orbital shaking at 450 rpm the lymphBUT was well formed and 200 µL of PBS were disposed. The formed lymphBUT was resuspended gently in the remaining PBS, thus leading to smaller lymphBUTs of sizes between 50 – 100 µm later acting as T cell activation zones. In a second step, 1 mol% biotin scaffolding (non‐functionalized) dsLBs were added with the appropriate streptavidin concentration of 0.40:1 biotin/streptavidin ratio to form a connecting scaffold around the already existing, smaller T cell activating lymphBUT zones.

Vise versa to the heterotypic lymphBUTs, reverse heterotypic lymphBUTs were formed to investigate the impact on the activation zone and scaffold density on the activation dynamics and phenotyping of human CD8^+^ T cells. For this, the activation zones were built from 1% biotin dsLBs with a high antibody functionalization surrounded by 5 mol% biotin scaffolding (non‐functionalized) dsLBs.

In contrast to the heterotypic lymphBUT architectures, homogenous lymphBUTs were formed by pre‐mixing 5 mol% biotin activating (functionalized) and 1 mol% biotin scaffolding (non‐functionalized) dsLBs connection with streptavidin of a total biotin/streptavidin ratio of 0.40:1.

The ratios of immune stimulating dsLBs to connective scaffold dsLBs were varied (75/25, 50/50, and 25/75). The total dsLB number was kept at ≈2.7 million dsLBs per lymphBUT.

### LymphBUT Formation Based on Silica Beads

To compare the lymphBUT formation from dsLBs with stiffer beads, silica microspheres (Bangs Laboratories Inc., USA) with a diameter of 3.94 µm were covered with a lipid bilayer membrane identical to the dsLB membrane. For that 25 µL of silica beads were resuspended in PBS and centrifuged at 10 000 xg for 30 s. The washing step was repeated a second time, and the beads were resuspended in 100 µL PBS before incubating for 2 min with 50 µL SUVs containing 20 mol% EggPG, 5 mol% 18:1 MPB PE, 5 mol% 18:1 Biotinyl PE, 1 mol% LissRhodamine B‐PE, and 69 mol% EggPC (all Avanti Polar Lipids, USA). After the SUV incubation the silica beads were washed twice and resuspended in 500 µL PBS. The wells of a 48‐well plate were prepared with 400 – 450 µL of PBS supplemented with streptavidin (1:0.10, 1:0.40, and 1:1.60 streptavidin/biotin ratio). The beads shook overnight at 400 µL in an orbital motion. As controls the silica beads shook overnight without streptavidin and were incubated overnight with streptavidin without orbital shaking.

### Stereomicroscopy Imaging of LymphBUTs

For analyzing the self‐assembly process of lymphBUTs with 1 mol% or 5 mol% biotin (Avanti Polar Lipids, USA) in the dsLB membrane and different streptavidin (Invitrogen, USA) concentrations in the PBS environment, lymphBUTs were produced as just described. They were visualized in the 48‐well plate by bright field images acquired with a LEICA DFC450 stereo microscope (Leica Microsystems, Germany) and a 2x (PLANAPO 2.0x/39, Leica Microsystems, Germany) or a 5x objective (PLANAPO 5.0x/0.5, Leica Microsystems, Germany). The images were analyzed with the ImageJ software (NIH, USA) by background subtraction of the LUT inverted image, a global‐threshold segmentation, fill hole function, watershed particle separation and automated particle detection. The parameter considered for the formation robustness of lymphBUTs was the mean intensity of the single lymphBUTs that was related to the density and therefore to the stability of the lymphBUTs and the robustness of the formation process.

### Laser Scanning Confocal Microscopy Imaging of LymphBUTs

The lymphBUTs comprising activation zones connected with a non‐functionalized dsLB scaffold was visualized using the confocal laser scanning microscope LSM 880 (Carl Zeiss AB) equipped with a 10x objective (EC “Plan‐Neofluar” 10x/0.30 M27, Carl Zeiss AG, Germany) and 405, 488, and 633 laser lines. To visualize the human CD8^+^ T cell that infiltrated the lymphBUT, the T cell nuclei were fixed with 2% PFA and stained with 6.7 nm Hoechst 33342 trihydrochloride (Thermo Fisher, Germany). The images were analyzed with the ImageJ software (NIH, USA) by z‐projection and background subtraction.

### Connection Area Analysis using LSCM

In order to compare the connection areas between single dsLBs, they were either equipped with 1 or 5 mol% biotin and 1% Cy5 in the dsLB membrane. Those dsLBs were then connected to lymphBUTs with Alexa Fluor 405 labeled streptavidin (Thermo Fisher, Germany) at a ratio of 1.40:1 biotin/streptavidin.

The resulting constructs were then carefully transferred to an 8‐well Nunc LabTeK glass bottom chamber slide filled with 200 µL PBS. The lymphBUTs were imaged with the laser scanning microscope LSM 880 (Carl Zeiss AB) equipped with a 63x immersion oil objective (Plan‐Apochromat 63x/1.4 oil DIC M27, Carl Zeiss AG, Germany) and 405 and 633 nm laser lines. The images were analyzed with the ImageJ software (NIH, USA) by background subtraction and manual selection of the connection areas.

### T Cell Migration within LymphBUTs

The migration behavior of CD8^+^ within lymphBUTs was investigated and analyzed using LSCM. For this 80 000 T cells were co‐cultivated for 72 h at 37 °C and 5 % CO_2_ within lymphBUTs in a round bottom 96‐well plate and fully supplemented RPMI 1640 medium. Before imaging, the cells were stained with 6.7 µm Hoechst 33342 trihydrochloride (Thermo Fisher, USA) according to manufacturer specifications and washed three times through a partial medium exchange. To prevent the lymphBUT from moving during microscopy, they were confined. Therefore two 12 mm round glass cover slides (Carl Roth GmbH + Co. KG, Germany) with a height of 100 µm were glued on the left and right edge of the well of a 6‐well plate (Thermo Scientific, USA) with two component silicone (Picodent, Germany) acting as spacers for the confinement. In between these spacers 100 µL of the sample, including the lymphBUT, were added, before a 24 × 24 square glass cover slide (Carl Roth GmbH + Co. KG, Germany) was glued on top of the two round cover slides with two component silicone, confining the lymphBUT. Afterward, 3 mL fully supplemented RPMI 1640 medium was added into the wells. The sample was incubated for 2–3 h at 37 °C before. The T cell migration within the lymphBUTs was imaged for 15 h every 2 min using the laser scanning microscope LSM 880 (Carl Zeiss AB) equipped with a 10x objective (EC “Plan‐Neofluar” 10x/0.30 M27, Carl Zeiss AG, Germany) and 405 and 647 laser lines. The migration was visualized and analyzed with the ImageJ software (NIH, USA) by background subtraction.

### Localization of T Cells in Heterotypic and Reverse lymphBUTs

To identify the localization of T cells and T cell clusters in immune stimulating reaction zones and connective scaffold scaffolding zones of lymphBUTs, co‐localization studies were performed. For this, heterotypic and reverse lymphBUTs, both consisting of 75% reaction zones and 25% scaffolding zones, were cultivated with 80 000 T cells in 96‐well flat bottom plates for 4 days. The CD8^+^ T cells were visualized by a 6.7 µm Hoechst 33342 trihydrochloride staining (Thermo Fisher, USA) and imaged with the confocal laser scanning microscope LSM 880 (Carl Zeiss AB) equipped with a 10x objective (EC “Plan‐Neofluar” 10x/0.30 M27, Carl Zeiss AG, Germany) and 405, 488, and 633 laser lines. The Mendels’ coefficient describing the co‐localization was analyzed using the ImageJ software (NIH, USA) and the Just Another Colocalization Plugin.^[^ ^42^ ^]^

### Mouse Lymph Node Isolation

Wild‐type (WT) C57BL/6N mice were purchased from Charles River. Mesenteric lymph nodes were isolated from 20–22‐week‐old mice for use in experiments. Animals were housed under standard conditions (22 °C, 50–60% humidity, and a 12 h light/dark cycle). All experimental procedures were approved by and conducted in accordance with the regulations of the State Office for Consumer Protection, Saarland (Landesamt für Verbraucherschutz; approval number: AZ 2.4.1.1 and 11/2021).

### Mechanical Properties of LymphBUTs and Naturals Mouse Lymph Nodes

The mechanical properties of lymphBUTs and natural mouse lymph nodes were determined by micro indentation. For this, mouse lymph nodes were isolated as described and dsLBs with 1 mol% and 5 mol% biotin in the membrane were connected with 0.40:1 biotin/streptavidin ratio and measured with the Microindenter G2 CellScale (CellScale biomaterials testing, Canada). To do so, the lymphBUTs and natural mouse lymph nodes were transferred to the CellScale fluid bath filled with 45 mL PBS on top of the testing anvil. The tungsten microbeam with a diameter of 0.0762 mm and a modulus of 411 000 MPa was equipped with a 1×1 mm square stainless‐steel platen functioning as cantilever. The mechanical properties of lymphBUTs natural mouse lymph nodes were measured by z compression with a compression magnitude of 10% of the lymphBUT height. The microbeam displacement was controlled in a ramp setting with a loading duration of 30 s, a holding duration of 5 s and a recovery duration of 30 s. The analysis is based on real time image tracking at a frequency of 5 Hz. The images were acquired using a USB digital camera with a zoom lens and XYZ automatized stage. The indentation force for lymphBUTs produced in two individual batches with 1 mol% and 5 mol% biotin in the dsLB membrane and two individual mouse lymph nodes was measured (n = 9). Scale bars in the images were calculated based on measuring the pixel aspect ratio with the 1 mm wide compression plate as the software does not read out direct pixel aspect ratios (1089 pixel = 1 mm).

### Integrating Metabolic Components into the LymphBUT

As many natural tissues show metabolic features, metabolic components were integrated into lymphBUTs in the form of enzyme loaded unilamellar vesicles (UVs). To produce the LUVs 5 mol% 18:1 Biotinyl PE (biotin), 1 mol% LissRhodamine B‐PE, Atto488‐PE and 94 mol% EggPC (all Avanti Polar Lipids, USA). The lipids were pre‐mixed and dried for 1 h using a vacuum pump. A sucrose (Sigma–Aldrich, UK) solution diluted to 285 mm with PBS and 2 000 U Glucose Oxidase from Aspergillus niger (Sigma–Aldrich, UK) per mL was added to the dried lipids and incubated for 15 min protected from light. After the incubation the lipids were resuspended and extruded using Nucleopore membrane of 1.0 µm pores (Cytiva, Germany). In order to get rid of the excess Glucose Oxidase, the UVs were diluted 1:200 with PBS and centrifuged at 10 000 xg for 6 min. The supernatant was deposed, and the pellet was resuspended in fresh PBS. The washing step was repeated three times. The LUVs were integrated into the dense 5 mol% biotin lymphBUT activation zones resulting in a total Glucose oxidation concentration in the lymphBUTs of 0.02, 0.20, and 2.00 U.

### Immune Cell Isolation and Cultivation

Primary human CD8^+^ T cells have mainly been used for functional assays with lymphBUTs. The T cells were isolated from leukapheresis reduction system chambers from healthy voluntary blood donors. Negative isolation was performed with commercial selection kits (RosetteSep Human CD8^+^ T cell Enrichment Cocktail, STEMCELL technologies, Germany) following the manufacturers instruction. The protocol has been adopted in order to isolate Peripheral Blood Mononuclear Cells (PBMCs) by omitting the negative selection. The Institute for Clinical Hemostaseology and Transfusion Medicine, Saarland University Medical Center provided the blood following ethics agreement number 34/23 (Ethikkommission Ärztekammer des Saarlandes). After the isolation the immune cells were frozen in RPMI 1640 w/ L‐Glutamine (VWR, Germany) medium supplemented with 40% fetal bovine serum (Gibco, Germany), 1% penicillin/streptomycin (Gibco, Germany), 1% non‐essential amino acids (Biowest) and 50 mm HEPES (Sigma–Aldrich, Germany) and additional 10% dimethyl sulfoxide (DMSO) (Sigma Aldrich, Germany) and stored at −80 °C until further use.

The primary human CD8^+^ and PBMCs were thawed one day before starting the experiment and incubated overnight. The cells were cultivated in RPMI 1640 w/L‐Glutamine (VWR, Germany) medium supplemented with 10% fetal bovine serum (Gibco, Germany), 1% penicillin/streptomycin (Gibco, Germany), 1% non‐essential amino acids (Biowest, France) and 50 mm HEPES (Sigma–Aldrich, Germany) with 50 U mL^−1^ of the growth factor Interleukin 2 (IL‐2) in T 25 cell culture flasks at 37 °C and 5% CO_2_.

### T Cell Ex Vivo Activation and Expansion within LymphBUTs

The isolated primary human CD8^+^ T cells were co‐cultivated with lymphBUTs in round bottom 96‐well plates and fully supplemented RPMI 1640 medium. Therefore, different lymphBUT architecture, as described, were formed, washed, and transferred with 100 µL to the round bottom 96 well plates. Dispersed activating (high antibody functionalized) dsLBs and Dynabeads human T‐Activator CD3/CD28 beads (Gibco, Germany) used according to manufacturer suggestions served as controls.

Because the lymphBUTs cannot form in RPMI medium due to the supplementation of vitamins including biotin they were formed in PBS. Therefore, they need to be extensively washed with fully supplemented RPMI 1640 medium before transferring within 100 µL of medium to the round bottom 96‐well plate. Because of the lymphBUT size of 1–4 mm, 1/10 of the pipette tip needs to be cut to increase the diameter of the tip opening and not destroy the lymphBUT. 80 000 T cells were transferred to each well containing a lymphBUT leading to a total medium volume of 250 µL and IL‐2 concentration of 50 U mL^−1^. The wells at the outer border were filled with PBS in order to avoid evaporation and therefore biased results. The primary human CD8^+^ T cells were incubated with the lymphBUTs in fully supplemented RPMI 1640 medium at 37 °C and 5% CO_2_ for 4 days.

### Cytokine Analysis using an Automated ELISA System

In order to analyze T cells activation, expansion as well as T cell phenotyping, primary human CD8^+^ T cells were co‐cultivated with different lymphBUTs architectures in fully supplemented RPMI1640 medium for 4 days. After the incubation the 80 µL of supernatant were transferred to another 96 well plate and either used directly or frozen at −80 °C until further use. The cytokine concentrations of IL‐10, IL‐17A, and IFNγ 3rd gen were measured with the Ella Automated ELISA protein simple system (Biotechne, USA). The samples were prepared according to manufacturer instructions and analyzed with the Simple Plex Runner software (Biotechne, USA).

### CD8^+^ T Cell Activation Studies using Flow Cytometry

To further study primary human CD8^+^ T cell activation and immunosuppression signals as well as phenotype related surface receptors, the lymphBUTs hosting the CD8^+^ T cells were resuspended in order to isolate the T cells and the dsLBs, centrifuged at 300 xg for 5 min, the supernatant was disposed, and the cells were resuspended in PBS containing staining antibodies (**Table** **1**).

**Table 1:** List of antibodies against different immune cell surface receptors containing the clone, dilutions, and company.

Antibody against:	Clone	Company	Dilution
CD25	BC96	Biolegend, UK	1:400
PD‐1	NAT 105	Biolegend UK	1:400
CD8	REA715	Miltenyi Biotec	1:50
CD103	Ber‐ACT8	Biolegend, UK	1:400
CCR7	REA556	Miltenyi Biotec	1:50
CD45RO	REA611	Miltenyi Biotec	1:50
CD95	REA738	Miltenyi Biotec	1:50
CTLA4	REA1003	Miltenyi Biotec	1:50
CD4	REA623	Miltenyi Biotec	1:50

Immune cells were resuspended in a Albumin fraction V (BSA) (Sigma–Aldrich, Germany) solution (1% BSA in PBS) to block unspecific binding and incubated for 30 min before they samples were centrifuged and the supernatant disposed again. The cell‐dsLB mix was resuspended in the antibody solution for incubated for 1 h at RT protected from light. After incubation the cells were washed twice and resuspended in PBS containing 2% PFA (Sigma–Aldrich, Germany) for 30 min at RT protected from light in order to fix the T cells. The cells were than washed again to remove the remaining PFA and resuspended in PBS containing 6.7 nm Hoechst 33342 trihydrochloride (Thermo Fisher, Germany) for staining the T cell nucleus for 20 min protected from light (for CD25 and PD‐1). Finally, the cells were washed once more and resuspended in PBS + 1% BSA. To avoid bleed thought compensation has been performed using the IgG compensation beads (Miltenyi Biotec). For the quantification of the surface marker the Attune NxT Flow Cytometer and the Attune Software (Thermo Fisher, Germany) were used. The flow cytometer is equipped with 405, 488, 561, and 637 nm laser lines. For the analysis, a minimum of 10 000 events were considered and analyzed with the FlowJo V.10 software (FlowJo LLC, USA).

### Analyzing the Immuno‐Phosphorylation Pattern via Proteom Profiler

To evaluate differences in intracellular signaling between Dynabeads, dispersed dsLBs (2 dsLBs:1 T cell) and 75‐heterogen lymphBUTs the relative phosphorylation of 59 receptors was analyzed. For this 100 000 human CD8^+^ T cells were co‐cultivated with the mentioned activation methods for 4 days. The cell samples were prepared according to the manufacturer protocol of Proteom Profiler Human Phospho‐Immunoreceptor Array and chemiluminescence was measured with the LI‐COR ODYSSEY *M* (LI‐COR Biosciences GmbH, Germany). The analysis was performed with the Empira Studio 3.2 (LI‐COR Biosciences GmbH, Germany).

### Validating the Human CD8^+^ T Cell Proliferation via Flow Cytometry

In order to compare the fraction of proliferated T cells, human CD8^+^ T cells were stained with CellTrace CFSE (Thermo Fisher, Germany). For this, CellTrace CFSE was dissolved in DMSO to a stock concentration of 5 mm and then further diluted in pre‐warmed (37 °C) PBS to 5 µm. After T cells thawing and overnight incubation the medium was disposed. The T cells were resuspended in the PBS / CellTrace CFSE mix and incubated at 37 °C for 20 min before they were centrifuged at 300 g for 5 min and resuspended in fully supplemented RPMI 1640 medium. This washing step was repeated a second time, and the cell concentration was adjusted to 80 000 T cells/well. The stained T cells were co‐cultivated with different lymphBUT conditions, dispersed dsLBs (2 dsLBs:1 T cell) and Dynabeads for 4 days and then measured by flow cytometry equipped with 405 and 561 nm laser lines. For the analysis a minimum of 10 000 events were considered and analyzed with the FlowJo V.10 software (FlowJo LLC, USA).

### Data Processing and Statistical Analysis

Graphs were plotted with GraphPad Prism 8 as mean ± SD of technical and biological replicates. Statistical analyses were performed with the in‐build GraphPad Prism 8 software function. The applied statistical analysis were noted for the individual figures in the figure legends. Schematic illustrations were created with BioRender.com.

## Conflict of Interest

The authors declare no conflict of interest.

## Supporting information



Supporting Information

Supplemental Movie 1

## Data Availability

The data that support the findings of this study are available from the corresponding author upon reasonable request.
