# Supplementary material for: Synthetic Cell‐Based Tissues for Bottom‐Up Assembly of Artificial Lymphatic Organs
Source: Adv Healthc Mater. 2025 Oct 23;15(5):e03498. doi: 10.1002/adhm.202503498 (PMC12864575; doi:10.1002/adhm.202503498)
Supplement: Supplementary file 1 — Supporting Information [file ADHM-15-0-s001.docx]

**Supplementary figures
Synthetic cell-based tissues for bottom-up assembly of artificial lymphatic organs**

**Anna Burgstaller^1,2,3^, Tamara Nink**^1^**, Niklas Walter**^1^**, Erick Angel Lopez Lopez ^1^, Hsin-Fang Chang ^4^, Oskar Staufer^1,2,3,5*^**

1 INM – Leibniz Institute for New Materials Campus D2 2, 66123 Saarbrücken, Germany

2 Helmholtz Institute for Pharmaceutical Research Saarland Helmholtz Center for Infection Research Campus E8 1, 66123 Saarbrücken, Germany

3 Saarland University, Center for Biophysics, Campus Saarland, 66123 Saarbrücken, Germany

4 Saarland University, Centrum für Integrative Physiologie und Molekulare Medizin (CIPMM), Gebäude 48, 66421 Homburg, Germany

5 Max Planck Bristol Centre for Minimal Biology Cantock’s Close, Bristol BS8 1TS, UK

*Corresponding author: [oskar.staufer@leibniz-inm.de](mailto:oskar.staufer@leibniz-inm.de)


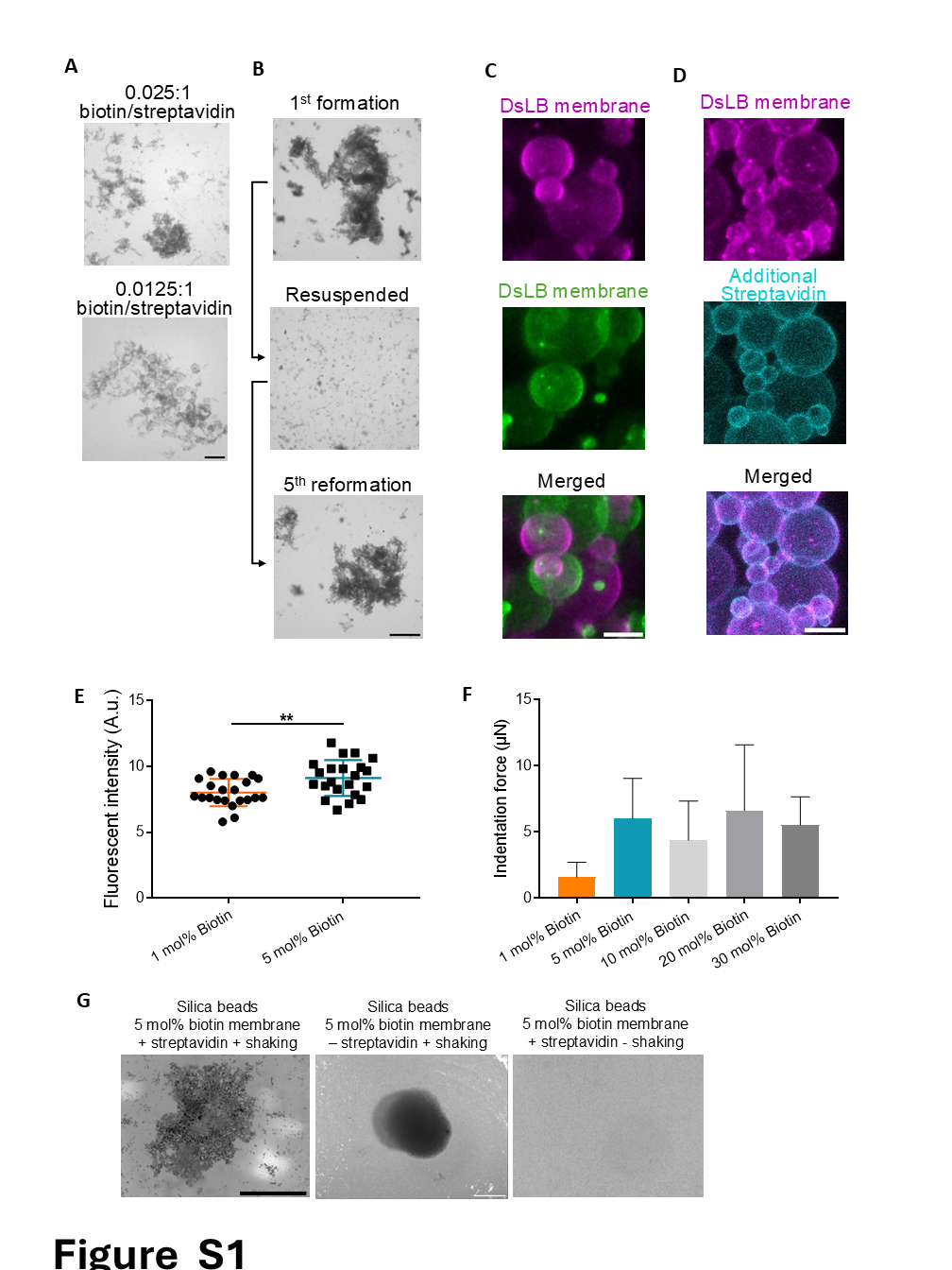


**Fig. S 1 LymphBUT formation** **A)** Stereo microscopy image of lymphBUTs formed with biotin/streptavidin ratios of 0.025:1 and 0.0125:1. Scale bar is 1 mm. **B)** Stereo microscopy images showing the re-formation property of lymphBUT after resuspension over 5 cycles. Scale bar is 1 mm. **C)** Representative confocal z-projection of homogenous lymphBUTs formed from two dsLB fractions (magenta (Rhodamine B) and green (Atto488) lipids) after mechanical disassembling and reformation. Scale bar is 5 µm. **D)** Representative confocal maximal z-projection of lymphBUTs reformed after mechanical rupturing with additional AlexaFluor405-labeled streptavidin. Scale bar is 5 µm. **E)** Comparison of fluorescent intensity of lymphBUTs made from dsLBs with 1 mol% and 5 mol% biotin in the membrane (n = 22 connection points). **F)** Comparison of maximal indentation force required to compress 10 % of total height of lymphBUTs made from dsLBs with 1, 5, 10, 20 and 30 mol% biotin in the dsLB membrane (n > 3 lymphBUTs). **G)** Silica beads surrounded by a lipid bilayer as used for dsLBs interconnected via 5 mol% biotin/streptavidin bond at a ratio of 100:1 with control experiments of lipid membrane coated, shaking silica beads without streptavidin and lipid membrane coated silica beads with streptavidin without shaking. Scale bar is 1 mm. Results are shown as mean +/- SD. P values were calculated using an unpaired two-tailed student t test. ** p<0.01

**
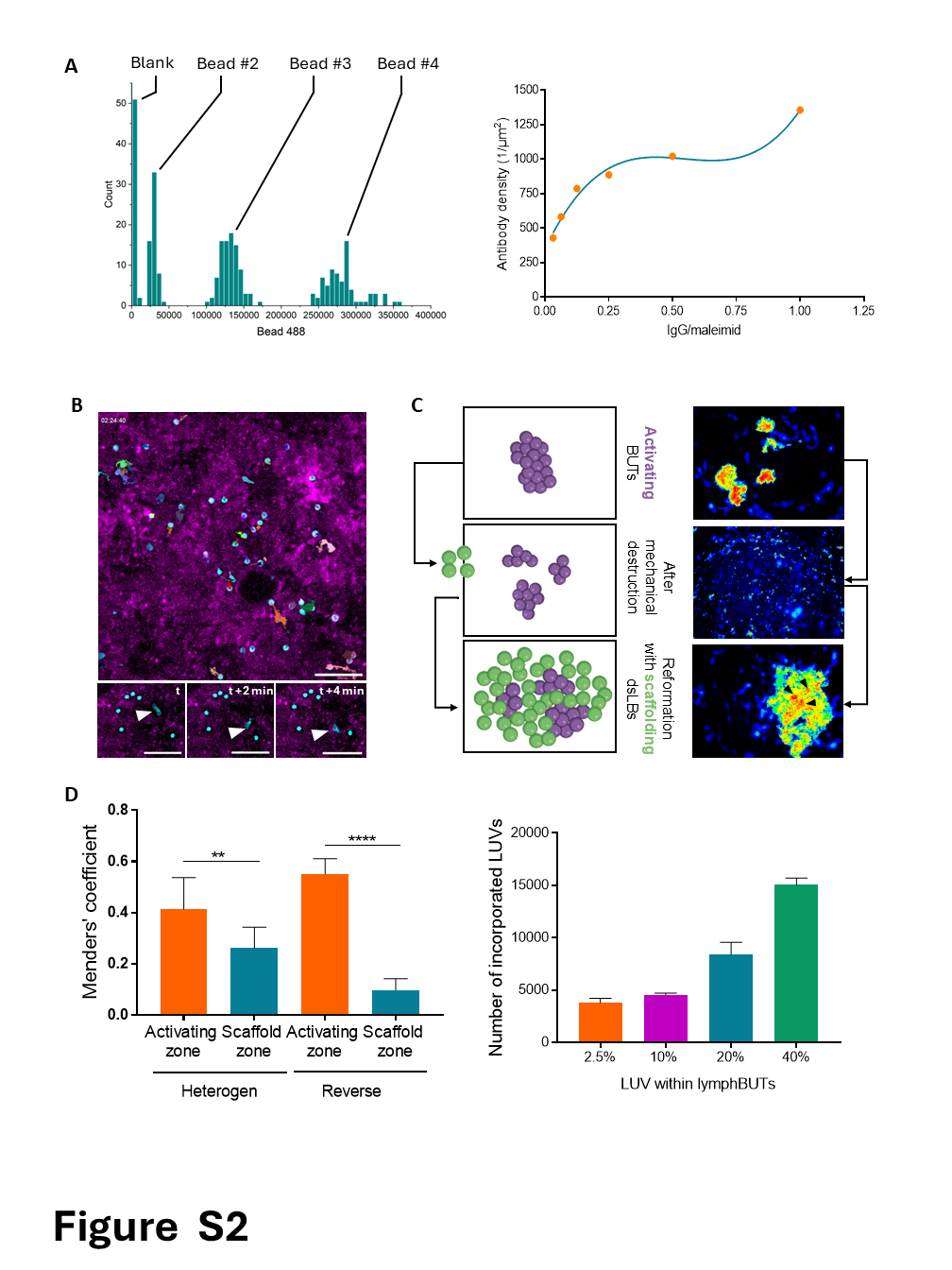
**

**Fig. S 2 Functional and structural lymphBUT properties impacting T cell location and migration A)** Fluorescent intensity quantification using MESF calibration beads for quantifying IgG density on dsLBs. **B)** Representative CD8^+^ T cells (cyan) tracking in a 5 % lymphBUT (magenta) and zoom in to morphologically changing T cell nucleus at three timepoints (white arrow). Scale bars are 50µm. Exemplary traces of individual cells are marked in color in the upper image. **C)** Sequential self-assembly process for heterotypic lymphBUT formation presented as illustrating scheme and representative microscopy images. **D)** Co-localization analysis of T cells in reaction or scaffold zones in heterogeny and reverse lymphBUTs. Results are shown as mean +/- SD from n > 10 spots of n > 4 lymphBUTs **E)** Number of incorporated LUVs in relation to the initially added concentration analyzed via confocal imaging. P values were calculated using an unpaired two-tailed student t test. ** p<0.01, **** p<0.0001


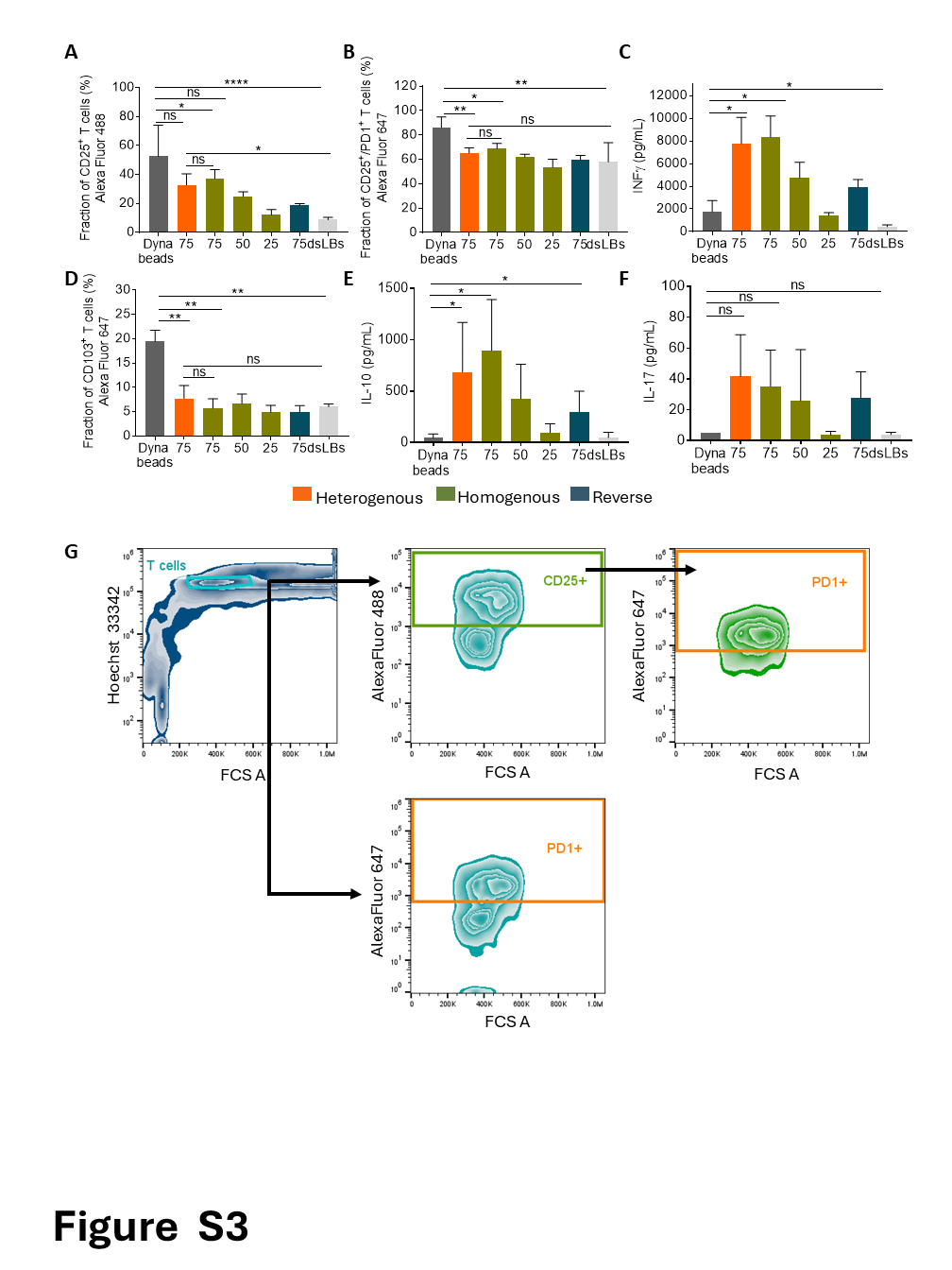


**Fig. S 3 Activation and immunosuppression profile of activated T cells depending on the lymphBUT architectures**
**A) and B)** Flow cytometry quantification of CD25^+^ CD8^+^ T cell population (A) CD25^+^/PD-1^+^ T cell subpopulation (B). **C)** Quantification of INFγ cytokine release via an automated ELISA. **D), E) and F)** Phenotyping of human CD8^+^ T cells by quantifying the CD103 expression via flow cytometry (F) as well as analyzing the IL-17 (F) and IL-10 (H) cytokine profile. Comparison of heterogenous (orange), homogenous (green) and reverse (blue) lymphBUTs with varying ratios of activating and non-activating dsLBs (75 %, 50 %, 25 %) displaying a high anti-CD3 and anti-CD28 concentration with Dynabeads and dispersed activating dsLBs without scaffolding dsLBs. **G)** Flow cytometry gating strategy exemplary for the Dynabead control. Results are shown as mean +/- SD of two donors n > 2. P values were calculated using two-tailed t test. ns = not significant p>0.05, * p<0.05, ** p<0.01, **** p<0.0001


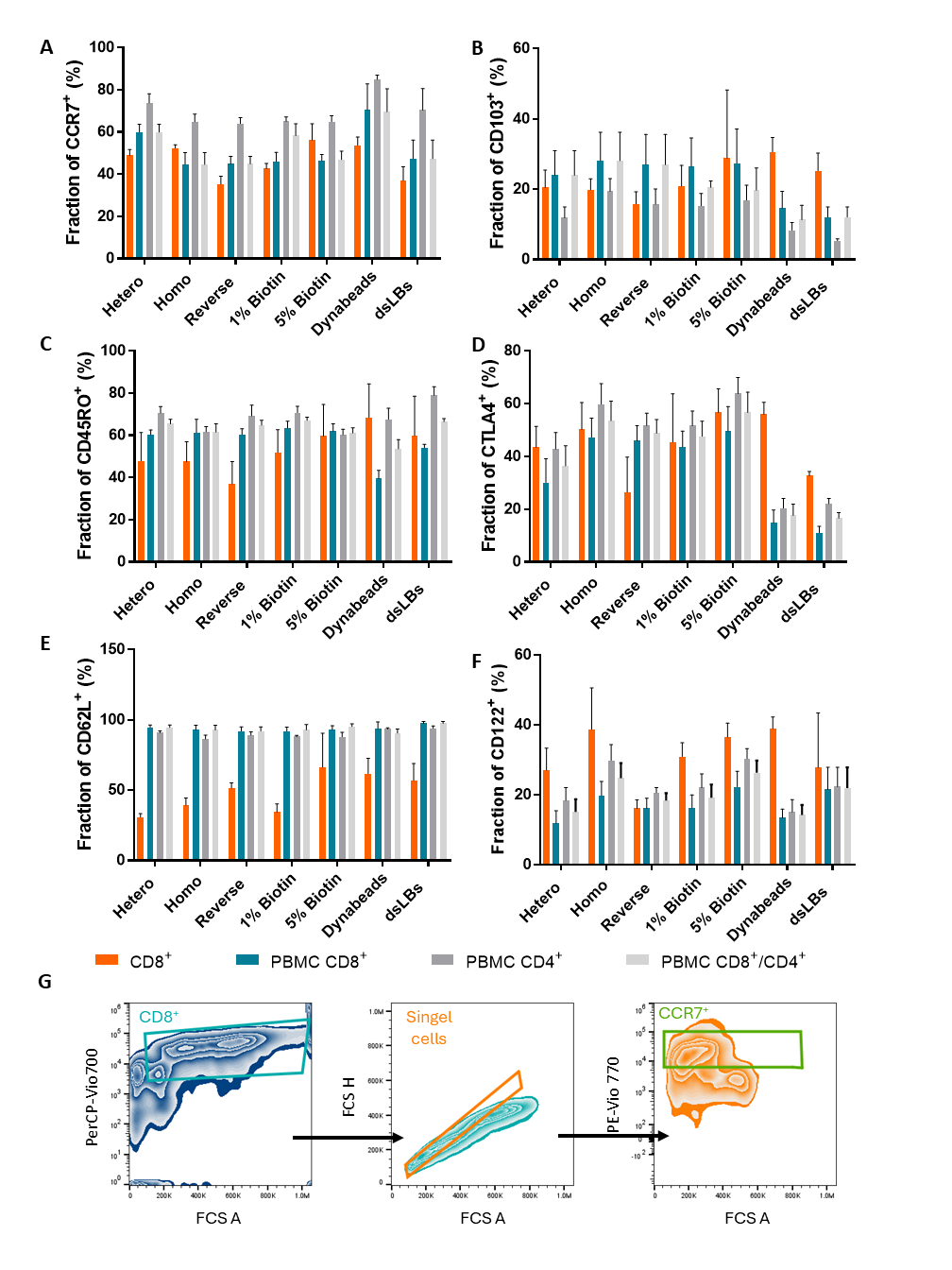


**Fig. S 4 T cell phenotyping** Flow cytometry quantification of **A)** CCR7, **B)** CD103, **C)** CD45RO, **D)** CTLA4, **E)** CD62L and **F)** CD122 of separated CD8^+^ T cells and CD8^+^ or CD4^+^ T cell isolated and incubated / activated within a PBMC mix as well as a combination of CD8^+^/CD4^+^ T cells form the PBMC mix. **G)** Flow cytometry gating strategy exemplary for the Dynabead control.
